# Supplementary material for: Is spatial exposure to heritage associated with visits to heritage and to mental health? A cross-sectional study using data from the UK Household Longitudinal Study (UKHLS)
Source: BMJ Open. 2023 Mar 28;13(3):e066986. doi: 10.1136/bmjopen-2022-066986 (PMC10069496; doi:10.1136/bmjopen-2022-066986)
Supplement: Supplementary data [file bmjopen-2022-066986supp001.pdf]

Supplemental table 1. EIMD Income Quintile descriptive – population, geographical area, heritage.

EIMD Income	Population (Mean, SD)	Area km² (Mean, SD)	Listed buildings (N)	Historic Parks/ Gardens (N)	Scheduled Monuments (N)	All sites (N)
**1 (most deprived)**	11,075,909 (1,686, 331.36)	3,428 (0.52, 1.19)	19,880	203	277	20,360
**2**	11,149,857 (1,697, 362.87)	7,547 (1.15, 3.71)	41,189	292	913	42,394
**3**	11,016,312 (1,677, 360.18)	26,114 (3.98, 12.42)	75,084	470	4,170	79,724
**4**	10,820,663 (1,647, 355.98)	50,252 (7.65, 20.69)	125,462	790	7,904	13,4156
**5 (least deprived)**	10,723,586 (1,632, 407.47)	43,089 (6.56, 15.89)	113,923	927	5,893	120,743
**Total**	54,786,327 (1,668, 365.19)	130,432 (3.97, 13.34)	375,538	2,682	19,157	397,377

Supplemental table 2. Complete case sample descriptive (percentage by each category)

(base n=30420)

**Age**	<20 years old	6.1
	20-29 years old	12.6
	30-39 years old	15.9
	40-49 years old	18.9
	50-59 years old	16.9
	60-69 years old	15.3
	70+ years old	14.4
	Missing	0
**Sex**	Male	44.6
	Females	55.4
	Missing	0

**EIMD income**	Q1 (most deprived)	20.5
	Q2	19.3
	Q3	20.6
	Q4	20.7
	Q5 (least deprived)	18.9
	Missing	0
**Ethnicity**	White	82.5
	Other	17.5
	Missing	21
**Education**	Degree or higher	36.4
	GCSEs/A-levels	41.8
	Other qualification	9.6
	No qualification	12.3
	Missing	54
**Job type (NS-SEC)**	Managerial, admin & professional	24.7
	Intermediate occupations	13.7
	Routine & manual occupations	19.8
	Not in paid employment	41.9
	Missing	341
**Housing tenure**	Owner occupier	71.3
	Social renter	16.7
	Private renter	12.1
	Missing	499
**Long-term illness/mobility**	No long-term illness or mobility issue	53.0
	Long-term illness	14.8
	Long-term illness and mobility issue	32.2
	Missing	11

**Marital/cohabitating status**	Single	21.8
	Married/cohabiting	63.7
	Divorced/separated	8.6
	Widowed	5.9
	Missing	70
