# Supplementary material for: Is spatial exposure to heritage associated with visits to heritage and to mental health? A cross-sectional study using data from the UK Household Longitudinal Study (UKHLS)
Source: BMJ Open. 2023 Mar 28;13(3):e066986. doi: 10.1136/bmjopen-2022-066986 (PMC10069496; doi:10.1136/bmjopen-2022-066986)
Supplement: Author's manuscript [file bmjopen-2022-066986.draft_revisions.pdf]

BMJ Open is committed to open peer review. As part of this commitment we make the peer review history of every article we publish publicly available.

When an article is published we post the peer reviewers' comments and the authors' responses online. We also post the versions of the paper that were used during peer review. These are the versions that the peer review comments apply to.

The versions of the paper that follow are the versions that were submitted during the peer review process. They are not the versions of record or the final published versions. They should not be cited or distributed as the published version of this manuscript.

BMJ Open is an open access journal and the full, final, typeset and author-corrected version of record of the manuscript is available on our site with no access controls, subscription charges or pay-per-view fees (http://bmjopen.bmj.com).

If you have any questions on BMJ Open's open peer review process please email info.bmjopen@bmj.com

BMJ Open

Heritage exposure and associations with reported visits to heritage, and with mental health, in the UK Household Longitudinal Study.

Journal:	*BMJ Open*
Manuscript ID	bmjopen-2022-066986
Article Type:	Original research
Date Submitted by the Author:	27-Jul-2022

Complete List of Authors:	Macdonald, Laura; University of Glasgow MRC/CSO Social and Public Health Sciences Unit, MRC Social and Public Health Sciences Unit; University of Glasgow MRC/CSO Social and Public Health Sciences Unit, Nicholls, Natalie; University of Glasgow MRC/CSO Social and Public Health Sciences Unit<br>Gallou, Eirini; Historic England, Policy and Evidence<br>Monckton, Linda; Historic England, Policy and Evidence<br>Mitchell, Richard; University of Glasgow MRC/CSO Social and Public Health Sciences Unit

Keywords:	MENTAL HEALTH, STATISTICS & RESEARCH METHODS, PUBLIC HEALTH

SCHOLARONE™
Manuscripts

I, the Submitting Author has the right to grant and does grant on behalf of all authors of the Work (as defined in the below author licence), an exclusive licence and/or a non-exclusive licence for contributions from authors who are: i) UK Crown employees; ii) where BMJ has agreed a CC-BY licence shall apply, and/or iii) in accordance with the terms applicable for US Federal Government officers or employees acting as part of their official duties; on a worldwide, perpetual, irrevocable, royalty-free basis to BMJ Publishing Group Ltd ("BMJ") its licensees and where the relevant Journal is co-owned by BMJ to the co-owners of the Journal, to publish the Work in this journal and any other BMJ products and to exploit all rights, as set out in our licence.

The Submitting Author accepts and understands that any supply made under these terms is made by BMJ to the Submitting Author unless you are acting as an employee on behalf of your employer or a postgraduate student of an affiliated institution which is paying any applicable article publishing charge ("APC") for Open Access articles. Where the Submitting Author wishes to make the Work available on an Open Access basis (and intends to pay the relevant APC), the terms of reuse of such Open Access shall be governed by a Creative Commons licence – details of these licences and which Creative Commons licence will apply to this Work are set out in our licence referred to above.

Other than as permitted in any relevant BMJ Author's Self Archiving Policies, I confirm this Work has not been accepted for publication elsewhere, is not being considered for publication elsewhere and does not duplicate material already published. I confirm all authors consent to publication of this Work and authorise the granting of this licence.

For peer review only

Heritage exposure and associations with reported visits to heritage, and with mental health, in the UK Household Longitudinal Study.

Laura Macdonald [a], Natalie Nicholls [a], Eirini Gallou [b], Linda Monckton [b], Richard Mitchell [a].

a. MRC/CSO Social and Public Health Sciences Unit, University of Glasgow, Glasgow, UK

b. Policy and Evidence, Historic England, London, UK

Correspondence to:

Mrs Laura Macdonald,

Places and Health Programme,

MRC/CSO Social and Public Health Sciences Unit,

University of Glasgow.

laura.macdonald@glasgow.ac.uk

For peer review only

Abstract

Objectives: Existing research highlights the beneficial nature of heritage engagement for mental health, but engagement varies geographically and socially, and few studies explore spatial exposure (i.e. geographic availability) to heritage and heritage visits. Our research questions (RQ) were 'does spatial exposure to heritage vary by area income deprivation?' (RQ1), 'is spatial exposure to heritage linked to visiting heritage?' (RQ2), and 'is spatial exposure to heritage linked to mental health?' (RQ3). Additionally, we explored whether local heritage is associated with mental health regardless of the presence of green space.

Design: Data collected January 2014-June 2015 via the UK Household Longitudinal Study (UKHLS) wave 5. Our study is cross-sectional.

Setting: General population, England.

Participants: 30,431 adults (16 years+) (13,676 males, 16,755 females). Participants geocoded to Lower Super Output Area (LSOA) 'neighbourhood' and 'English Index Multiple Deprivation' (EIMD) 2015 Income score.

Primary and secondary outcome measures: LSOA-level heritage exposure and green space exposure (i.e., population and area densities, percentage land cover); heritage site visit in past year (binary: no, yes); mental distress (General Health Questionnaire-12, binary: less distressed 0-3, more distressed 4+).

Results: Heritage varied by deprivation, the most deprived areas (Income Quintile (Q)1:1.8) had fewer sites per 1000 population than the least deprived areas (Q5:11.1) (p<0.01). Compared to those with no LSOA-level heritage, those with heritage exposure were more likely to have visited a heritage site in the past year (OR: 1.12 (95%CI: 1.03-1.22)) (p<0.01). Amongst those with heritage exposure, visitors to heritage had a lower predicted probability of distress (0.171 (95%CI: 0.162-0.179)) than non-visitors (0.238 (95%CI: 0.225-0.252)) (p<0.001).

Conclusions: Our valuable research contributes to evidence for the wellbeing benefits of heritage and is highly relevant to the government's levelling-up heritage strategy. Our findings can feed into schemes to tackle inequality in heritage exposure to improve both heritage engagement and wellbeing.

Keywords: heritage, GIS, inequalities, mental health.

For peer review only

Strengths and limitations of this study

- Our paper is the first to explore associations between spatial exposure to heritage, and visits to heritage, and mental health, within relatively small areas across England.
- We use a sample of adults from across England, weighted to ensure representativeness to the wider population of England.
- We use a variety of LSOA-level measures to capture exposure to heritage, i.e. population and area densities, percentage of land cover, and percentage of sites as 'at risk'.
- We explore whether mental health benefits of heritage remained regardless of the presence of local green space, providing a unique strand to this research.
- Our study may be limited in that we do not know whether UKHLS respondents visit heritage sites within their local area or further afield.

Introduction

Population health research has paid much attention to the health benefits of exposure to natural environments [1]. Regular contact with green space has been shown to benefit physical health, e.g. through reduced risk of hypertension, stroke, cardiovascular disease and asthma, and improved immune function [2]. There is stronger evidence for mental health benefits of time spent in green and natural space, such as improved cognitive function and brain activity [1], greater social cohesion, and sense of community, relaxation and restorative psychological effects, and lower levels of depression, anxiety and stress [3].

A much smaller evidence base suggests that visiting heritage may also benefit mental health and wellbeing. In this study, we define heritage as a building, monument, site or area classified as having 'a degree of significance' worthy of consideration in planning related decisions, due to its architectural or historic interest [4]. Heritage assets may be 'designated' by the Secretary of State for Digital, Culture, Media and Sport (DCMS) as advised by Historic England, or recognised for their significance by local planning authorities, while 'World heritage sites' are further recognised for their international value by bodies such as UNESCO. People who frequently visit heritage sites report higher life satisfaction [5-7], enhanced levels of self-esteem and happiness [8], and lower levels of mental distress [9]. Residents of places with greater numbers of heritage sites have a stronger 'sense of place' [10] and higher levels of place attachment have been associated with better quality of life [11], and 'social wellbeing' [12]). Studies have also shown that engaging with heritage can be psychologically restorative [13], and that visits to cultural heritage sites can reduce stress [14]. The

For peer review only

denial of access to heritage during the Covid-19 lockdowns also provided evidence for its benefits. Awareness of the value of heritage to mental wellbeing was reportedly reinforced by being unable to access it [15].

One problem with studies of both green space and heritage is that they do not often allow for each other, or acknowledge the overlap between them. Historic parks and gardens are 'cultural assets' in that they are commonly designed or created by and for people with a purpose. They are often cultural landscapes in their own right, but many parks and gardens contain historic buildings or have historic value, and many monuments and buildings are close to or surrounded by green spaces. Furthermore, the parts of towns and cities that contain more (or less) heritage might well also contain more (or less) green space and trying to 'untangle' the contributions of each environment to health and wellbeing is a difficult task. Overall though, evidence suggests that exposure to heritage has the potential to benefit health, and in particular, mental health.

Despite the potential benefits, visits to heritage are unequal. People living in more deprived areas are less likely to engage in the arts, culture and heritage regardless of personal socio-economic status [9]. In the UK in 2019/2020, a higher proportion of adults resident in the least deprived areas (83%) had visited sites in the past year than those resident in the most deprived (50%) [16]. Heritage engagement levels vary by individual characteristics too with, e.g., those in employment, and in higher socio-economic groups, more likely to visit [17]. Geographical availability of cultural assets, such as museums and libraries is associated with visits or engagement [18], but availability is unequal across the UK with less deprived areas showing better access [19]. In this study, we refer to this geographical availability factor as 'exposure.' There is surprisingly little evidence about exposure to heritage, about how it interacts with the socio-economic characteristics of the population and whether it is implicated in any health benefits. Such evidence is important to both agencies concerned with improving and equalising population health, and those seeking to understand the value and potential of heritage. If levels of exposure make a difference for both engagement and health, there is potential for place-based schemes to improve and equalise it.

To address this gap, in this paper we explore whether spatial exposure to designated heritage (i.e. listed buildings, scheduled monuments and historic parks/gardens) varies by area income deprivation within England, and whether spatial exposure to heritage is linked to visits to heritage (unadjusted and adjusted for socio-demographic factors). We then explore whether exposure to heritage is associated with mental health (GHQ12) among all residents, and those who are visitors and non-visitors (controlling for socio-demographic factors and other green space). Additionally, we explore 'built' heritage, specifically, to understand whether exposure to listed buildings and

For peer review only - http://bmjopen.bmj.com/site/about/guidelines.xhtml

scheduled monuments is associated with mental health benefits beyond those associated with exposure to green space. We consider whether the physical presence of heritage in an area could improve psychological wellbeing among those living in proximity to it, and disentangle the potential overlap in benefits related to heritage exposure and to green space exposure. Our research is highly valuable and timely, with, in the 2022 Levelling Up paper, the UK government showing a commitment to tackling socio-spatial inequalities in heritage access and engagement [20].

In summary, our three research questions (RQ) are: RQ1 'does spatial exposure to heritage (and heritage 'at risk') vary by area deprivation?', RQ2 'Is spatial exposure to heritage linked to visiting heritage?', and RQ3 'Is spatial exposure to heritage (i.e., any, or 'built' heritage specifically) linked to mental health among heritage visitors and non-visitors?'.

Methods

To answer our research questions, we needed four data components describing: (i) local area population, context and socio-economic situation; (ii) spatial exposure to heritage; (iii) spatial exposure to other green spaces; and (iv) individual level visits to heritage, health, and socio-demographic covariates. These data needed a common spatial framework to represent, and georeferenced individuals (i.e. residential location related to a ground system of geographic coordinates) to, the 'local area' or 'neighbourhood'. The spatial framework was Lower Super Output Area (LSOA) (as individual addresses or postal codes not available). LSOAs are small area units created for collecting and reporting statistics about the UK population [21]. LSOAs have a median area size of 45.5 hectares and median population size of 1,500 residents or 650 households. There are 32,844 LSOAs in England and they are commonly used as a proxy for 'neighbourhood'.

Data

(i) local area population, context and socio-economic situation

We obtained the English Index of Multiple Deprivation (EIMD) 2015 (income domain) ranks and quintiles [22] and 2015 mid-year population estimates [23] to describe LSOA population and socio-economic situation. An EIMD Income quintile descriptive is included within supplementary table 1 (including total and mean population/size per quintile).

(ii) and (iii) Spatial exposure to heritage and other green spaces.

Heritage data: We obtained spatial data on all nationally protected historic buildings and sites from Historic England for 2014 [4]. The National Heritage List for England originated in 1882 when the first powers of protection were established and became a statutory 'Listing' after World War Two (mid-

For peer review only

1940s). Currently, the List holds over 400,000 entries and draws together all scheduled monuments, listed buildings, registered landscapes, battlefields, and protected wrecks. Historic England continuously updates the list, curated from the DCMS [4]. In our analysis we included listed buildings (points only – in compliance with Ordnance Survey (OS) licence), scheduled monuments (polygons), and historic parks/gardens (polygons). We excluded battlefields and wreck-sites as the former are few, and in only specific geographical areas, and the latter may not be visible from land. Listed buildings are buildings (e.g. residences, farmhouses, churches etc.) recognised as being of special architectural or historic interest. Scheduled Monuments are historic sites of national importance that are legally protected, such as, Roman remains, burial mounds, castles, bridges, earthworks, etc. Historic parks/gardens are protected 'designed' landscapes, including a range of planned open spaces, such as public parks, cemeteries, private house grounds etc. The dataset also distinguished 'Heritage at Risk' (HAR) sites. HAR is an official description of a heritage asset added to the HAR register that is maintained by Historic England, and records the condition of designated heritage assets after assessment. Such sites are vulnerable to loss due to "neglect, decay or inappropriate development" and are in need of safeguarding and protection [24].

Other green space data: We obtained data on other green space for England from the OS Open Greenspace database. Data for 2014 were not available, with the closest temporal match being July 2017 [25]. These were vector data (scale 1:25000) including polygon boundaries of green spaces. We selected green spaces that we considered 'natural heritage', including: allotments and community growing spaces; cemeteries and other religious grounds; golf courses; and parks/gardens. We excluded spaces associated with sport/play: tennis courts; bowling greens; other sports facilities; playing fields; and play spaces. The historically significant parks/gardens included in the data from Historic England were likely to also feature in the OS data. To ensure we did not double count these, we overlaid the two datasets in a Geographic Information System (GIS), ArcMap 10.8.1, to check for and remove duplicates; any feature captured in the data from Historic England was removed from the OS green space data. The remaining features are referred to as 'other green space'. We obtained LSOA boundaries (2011) [26] and used the 'Overlay' 'Spatial Join' to link the points (or polygon centroids/centre points) in the heritage and other green spaces datasets to LSOA boundaries. If a site (polygon) was spread across more than one LSOA, it was linked to the LSOA in which its centroid was located. We then calculated a count of each heritage feature and a count of each other green space feature in each LSOA. We used the 'Overlay Intersect tool' to calculate the land cover of each heritage/green feature within each LSOA.

Exposure variables: We then used IBM SPSS statistics v28 to calculate four categorical exposure measures of heritage (at LSOA level). These were ‘presence or absence of heritage sites’, ‘heritage sites per 1000 population’, ‘heritage sites per kilometre squared (km²)’, and ‘percentage land as heritage’. Using these four measures allowed us to model whether certain ‘levels of’ heritage were important, and allowed us to account for variation in the geographical size of LSOAs and of historic parks/gardens and scheduled monuments. The per population and per area exposure variables were grouped into ‘none’, ‘low-medium levels (1-4 sites)’, and ‘higher levels’ (five or more sites)’, while the percentage land as heritage was grouped into ‘none’, ‘1-4%’, and ‘5% or more’. Due to no existing methodology on appropriate groupings of exposure we based our thresholds on the distribution of values and on percentile representations i.e., numbers roughly represented none, 25th-50th percentile, and 75th percentile, respectively. We also used the same method detailed above to create various exposure variables: ‘built’ heritage (i.e. listed buildings/scheduled monuments), scheduled monuments, historic parks/gardens, other green space (non-heritage), and any green space (heritage and non-heritage combined). We joined the area measures of heritage, ‘built’ heritage, other green spaces, and any green spaces to individuals via their LSOA of residence. UKHLS individuals’ LSOA codes were accessed via a UK Data Service special licence.

(iv) individual-level visits to heritage, health, and socio-demographic covariates

Visits to heritage: Individual-level data were drawn from wave five (2014) of the UK Household Longitudinal Study (UKHLS, 2021) (see supplementary table 2 for sample descriptive). Data were obtained via the UK Data Service. The UKHLS is a large panel survey, which has tracked around 31,000 people in England since 2009. We used wave five as it was the most recent wave with questions about visits to heritage. Respondents were asked how often they visited each of these heritage sites:-‘a city or town with historic character’, ‘a historic building’, ‘a historic place of worship’, ‘a historic park or garden, ‘a place of industrial history or historic transport system’, ‘a monument such as a castle, fort or ruin’, ‘a site of archaeological interest’ or a ‘sports heritage site’, in the past year. We did not know whether visits to heritage were in respondent’s LSOA or not.

Mental health measure and covariates: UKHLS carries a General Health Questionnaire (GHQ-12) subjective wellbeing measure, which we included as a measure of mental health. The GHQ-12 includes 12 questions evaluating general mental health functioning and distress. Responses use a 4-point scoring system, i.e. better than normal, same as usual, worse than usual, or much worse than usual, and responses are summed to a score varying from 0 to 12 [27]. We modelled the score as a binary variable ‘less distressed’ (0-3) and ‘more distressed’ (4+) (the range of GHQ-12 values was not appropriate for use as a numeric outcome). This binary cut-off was used previously green space and

For peer review only

mental health research [28]. Covariates were chosen based on their established associations with visits to heritage [17 29]. They were sex; age-group (10-year intervals); EIMD income (quintiles); household composition ('single adult', 'single adult with children under 16', 'two adults or more', 'two adults (or more) with children under 16'); ethnicity ('White', 'Black, Asian and Minority Ethnic (BAME)/mixed' (numbers did not permit more specific groupings)), highest education qualification ('degree/other higher degree', 'A-levels/GCSEs', 'other qualification', 'no qualification'), socio-economic status (NS-SEC - 'managerial/ professional', 'intermediate/small employment/own account', 'lower supervision/lower technician/semi-routine/routine', 'not in (paid) employment'), housing tenure ('owner occupier', 'social renter', 'private renter'), and long-standing health condition/mobility ('none', 'condition without limited mobility', 'condition with limited mobility').

Patient and public involvement: We did not involve study participants in the development of the research question, design and implementation of the study or interpretation of the results.

STROBE: Strengthening the Reporting of Observational studies in Epidemiology cross-sectional reporting guidelines were adhered to within this study [30].

Statistical analysis

Does spatial exposure to heritage vary by area deprivation (RQ1)? We compared median densities of sites (per 1000 population, per square kilometre (km^2)), and mean percentage land cover, across area income deprivation quintile, using the Kruskal Wallis test. We also calculated percentage of heritage sites as 'at risk' across area income deprivation quintile.

Is spatial exposure to heritage linked to visiting heritage (RQ2)? We used multilevel logistic regression to determine the association between heritage exposure measures and whether a respondent had visited a heritage site in the past year, controlling for individual socio-demographic covariates as listed above. The primary sampling unit of the survey was set as the random effect, weighted by the individual cross-sectional weights supplied by the data providers. For the variables of exposure with more than two levels, post-hoc multiple comparison tests were performed, and results also presented as predicted probability of visitation for each level. We undertook further similar modelling to explore whether visits to historic parks/gardens, and scheduled monuments, specifically, was related to exposure to these particular sites respectively.

Is spatial exposure to heritage linked to mental health (RQ3)? Multilevel logistic regression was used to determine the association between GHQ (as a binary outcome) and heritage exposure measures. The same heritage exposure variables as within the RQ2 models were included as independent variables, i.e. presence or absence of heritage sites, sites per 1000 population, sites per

For peer review only

km², and percentage land as heritage. We controlled for socio-demographic variables, 'visits to heritage' (as binary variable, 'no visits' and 'one or more visits') (to investigate a potential heritage exposure/visits interaction) and exposure to 'other green space' (to explore potential mental health benefits of heritage exposure regardless of green space). Furthermore, we repeated models with 'built' heritage only and controlled for 'all green space' (heritage and non-heritage combined).

The primary sampling unit was set as random effect and weighted with the cross-sectional individual weight. For the models, an interaction between visits and the heritage exposure variables was also tested. If not significant, the interaction was removed and the model rerun with the main effects only. Again, for variables of exposure with more than two levels, post-hoc multiple comparison tests were performed, and results presented as predicted probabilities of poorer mental health for each level of the factor. No specific treatment was given to missing data, a complete case approach was used. Multilevel models performed in STATA [31], with a global significance set at 5%.

Results

Heritage exposure varied by area deprivation in a manner dependent on the type of exposure (descriptive information on heritage numbers per quintile available within supplementary table 2) (RQ1). The most deprived areas (Q1) showed lower mean numbers of heritage per 1000 population, while Q4 and Q5 showed the highest mean numbers. This was evident for all four types of heritage analysed together and separately (see table 1). The pattern for mean numbers per area (i.e. per km²) across income deprivation was less clear. The most deprived areas (Q1) had the lowest numbers of listed buildings (9.08), scheduled monuments (0.06) and sites combined (9.23) (see table 1). Q2 had the highest mean number of listed buildings (12.92), and combined sites (13.10), while Q5 had the highest mean number of scheduled monuments (0.11). There appeared to be no statistically significant difference between areas of varying deprivation in mean numbers of historic parks/gardens per area. In tables 1-2, while the appropriate comparative test was used (i.e. Kruskal Wallis), means are presented instead of medians, as the latter were zero across all quintiles.

Table 1. Mean heritage sites (2014) per 1000 population, and per km², by EIMD Income (2015) quintile.

	**Listed buildings Mean number**		**Historic Parks/gardens Mean number**		**Scheduled Monuments Mean number**		**Total Mean number**	
**EIMD Income**	**per 1000 people**	**per km²**	**per 1000 people**	**per km²**	**per 1000 people**	**per km²**	**per 1000 people**	**per km²**
**1 (most deprived)**	1.79	9.08	0.02	0.09	0.03	0.06	1.84	9.23

For peer review only

**2**	3.73	12.92	0.03	0.09	0.08	0.09	3.84	13.10
**3**	6.80	12.27	0.04	0.09	0.37	0.10	7.21	12.46
**4**	11.39	9.57	0.07	0.09	0.72	0.09	12.18	9.75
**5 (least deprived)**	10.42	9.18	0.09	0.10	0.54	0.11	11.05	9.39
**Total**	6.83	10.61	0.05	0.10	0.35	0.09	7.23	10.8
**Kruskal Wallis**	P<0.001	P<0.001	P<0.001	P=0.909	P<0.001	P<0.001	P<0.001	P<0.001

With decreasing deprivation, there was an increase in mean percentage of land as historic parks/gardens (Q1:0.54 to Q5:1.21), and mean percentage of land as heritage combined (i.e. all types) (Q1:0.63 and Q5:1.41) (see table 2). The least deprived had more than twice the percentage of land designated to scheduled monuments (Q5:0.20%) than the most deprived (Q1:0.09%). Around 1% (n=4832) of listed buildings (n=2098), scheduled monuments (n=2639) and historic parks/gardens (n=95) were deemed as 'Heritage at Risk' (HAR). Over 2% in total were found in the most deprived fifth of areas, compared to 1% in Q5 (and between 1.1% and 1.4% in the other quintiles) (see table 3). The most deprived areas had the highest percentage of HAR sites across two of the three categories (2.0% of listed buildings, 21.7% of scheduled monuments). In general, the percentage of HAR listed buildings and scheduled monuments increased as areas become more income deprived. The least deprived areas (Q5) exhibited the highest percentage of historic parks/gardens as 'at risk', i.e. 4.1% of sites compared to 3.4% of sites in Q1.

Table 2. Mean percentage land cover as heritage (2014) by EIMD (2015) Income quintile.

	**Mean percentage land cover**		
**EIMD Income**	**Historic Parks/gardens**	**Scheduled Monuments**	**Total**
**1 (most deprived)**	0.54	0.09	0.63
**2**	0.71	0.12	0.83
**3**	0.75	0.16	0.91
**4**	0.98	0.15	1.13
**5 (least deprived)**	1.21	0.20	1.41
**Total**	0.84	0.14	0.98
**Kruskal Wallis**	P<0.001	P<0.001	P<0.01

Table 3. Percentage of heritage sites as 'Heritage at Risk' (2016) (HAR) by EIMD income (2015) quintile.

	**Percentage as HAR**

For peer review only

EIMD Income	Listed buildings	Historic Parks/gardens	Scheduled Monuments	Total
**1 (most deprived)**	2.0	3.4	21.7	2.3
**2**	0.8	2.7	16.4	1.1
**3**	0.6	3.8	15.4	1.4
**4**	0.4	3.0	13.2	1.2
**5 (least deprived)**	0.3	4.1	12.6	1.0
**Total**	0.6	3.5	13.8	1.2

Of 31,446 respondents, 59.9% had at least one heritage site within their LSOA, and 61% had visited heritage in the past year (n=19,232). Respondents with at least one heritage site of any type in their neighbourhood (LSOA) were significantly more likely to have visited a heritage site in the past year (Odds Ratio (OR) 1.12), than those with no sites (p=0.008) (see table 4) (RQ2). Those with five or more heritage sites per 1000 population, or per km², in their LSOA were most likely to have visited a heritage site (OR 1.16 and 1.14 respectively). A higher percentage of land as heritage did not increase the probability of visits to heritage (results not shown). The presence of scheduled monuments within respondents' neighbourhoods did not increase the probability of visiting monuments specifically, however with an increase in LSOA-level historic parks/gardens per area the likelihood of visiting such sites increased (OR 1.12 (95% CI 1.04-1.21), p<0.01) (results not shown).

Table 4. Odds of visiting heritage by exposure to heritage.

(Adjusted by household type, sex, age, income deprivation, ethnicity, education, socio-economic status, housing tenure & health condition/mobility)

		**Odds of visiting heritage**	
		**OR (95%CI)**	**p-value**
**Heritage (any)**	**None**	1.00	*0.008*
	**1+**	*1.12 (1.03-1.22)*	
**Heritage (any) per 1000 pop**	**None**	1.00	*0.014*
	**1-4**	*1.10 (1.00-1.20)*	
	**5+**	*1.16 (1.05-1.30)*	
**Heritage (any) per 1 sq km**	**None**	1.00	*0.027*
	**1-4**	*1.11 (1.01-1.22)*	
	**5+**	*1.14 (1.02-1.26)*	

For peer review only

As a main effect, there was no association between heritage exposure and psychological distress (GHQ-12), regardless of exposure measure used (p-values: 0.055 – 0.224) (RQ3). However, there was an interaction effect between heritage exposure and heritage visits (see table 5). Post-hoc testing (with Sidak adjustments for multiple comparisons) found the following associations: Amongst those with any heritage, visitors to heritage had a lower predicted probability of distress (0.171) compared to non-visitors (0.238) (Šidák adjusted p-value <0.001). This was also true when considering different levels of exposure, i.e. five or more sites per 1000 population (visitors: 0.165, non-visitors: 0.241, (Šidák p=0.008)), or per km² (visitors: 0.171, non-visitors: 0.246, (Šidák p=0.016)). We found similar results when 'built' heritage (i.e. listed buildings/scheduled monuments only) was included as the exposure variable (controlling for any green space). Amongst those with one or more built heritage sites, or with five or more per 1000 population, visitors had a lower predicted probability of distress – visitors: 0.171, non-visitors: 0.237 (p <0.001) and visitors: 0.165, non-visitors: 0.242 (p<0.01), respectively (results not shown).

Table 5. Predicted probabilities and 95% confidence intervals (CI) of poorer mental wellbeing (GHQ12) by heritage exposure and visits to heritage.

(Adjusted by marital, sex, age, income deprivation, ethnicity, education, socio-economic status, housing tenure, health condition/mobility, exposure to other green space & visits to heritage).

		**Non-visitors (CI)**	**Visitors (CI)**	**Interaction p-value**
**Heritage**	**None**	0.218 (0.204-0.233)	0.184 (0.174-0.195)	*p=0.005*
	**1+**	*0.238 (0.225-0.252)	*0.171 (0.162-0.179)	
**Heritage per 1000 population**	**None**	0.219 (0.205-0.234)	0.185 (0.174-0.196)	*p=0.011*
	**1-4**	0.235 (0.218-0.251)	0.173 (0.163-0.184)	
	**5+**	**0.241 (0.219-0.264)	**0.165 (0.152-0.177)	
**Heritage per area (1 km²)**	**None**	0.219 (0.205-0.234)	0.185 (0.174-0.195)	*p=0.014*
	**1-4**	0.230 (0.213-0.248)	0.169 (0.159-0.179)	
	**5+**	***0.246 (0.226-0.267)	***0.171 (0.158-0.184)	

Post-hoc multiple comparisons: *Difference p<0.001, **Difference p<0.01, ***Difference p<0.05.

For peer review only - http://bmjopen.bmj.com/site/about/guidelines.xhtml

Discussion

This is the first study to explore the spatial distribution of designated heritage sites across areas of varying deprivation in England, and associations between exposure and visits, and mental health. We found that, compared to wealthier areas, the poorest areas had fewer sites per population and, of these sites, a higher proportion were deemed 'at risk' (RQ1). Neighbourhood exposure to heritage was associated with visits to heritage (RQ2); however, the physical presence of neighbourhood heritage, on its own, was not associated with residents' mental health (RQ3). The combination of having heritage present in the neighbourhood *and* visiting heritage (whether within the neighbourhood or further afield), was associated with better mental health (RQ3).

Regarding the social-spatial patterning of heritage, wealthier areas in general had greater heritage per population/land cover, than poorer areas, however the distribution of heritage varied according to how heritage exposure was measured. Although poorer areas showed the lowest mean heritage sites per area (Q1: 9.2), wealthier areas followed closely (Q5: 9.4). This apparent similarity between Q1 and Q5 could be deceptive as wealthier areas are on average larger, and may have more heritage sites spread across larger geographical extents (LSOA size increase with increasing wealth). Indeed, greater distance to a local heritage site may be less of a spatial barrier to those on higher incomes, whom are more likely to own cars [32]. Little existing research investigates spatial distribution of heritage on a national scale. A US city-based study, found that higher income/educated neighbourhoods, had superior access to historical and heritage institutions [33]. The authors described spatial access to historical/heritage institutions as 'elitist' with unequal geographic distribution detrimental to poorer neighbourhoods. In our study, perhaps the spatial bias towards higher income areas is a result of heritage increasing overall area wealth. Indeed, conservation area homes in England benefit from price premiums and greater annual price growth [34], and those with the financial means will pay more to live in homes with historic features [35]. We also report that more heritage sites within poorer areas were 'at risk'. HAR sites are more likely to be in a state of disrepair, less aesthetically pleasing, and potentially unsafe, therefore their higher prevalence in poorer neighbourhoods may result in residents being less likely to engage with heritage. Other existing features of poorer neighbourhoods could compound this, such as higher environmental 'incivility' e.g. graffiti, litter, noise and air pollution [36], and vacant and derelict land [37]. It is conceivable that HAR's greater presence within lower income areas, is a result of inequity in the geographical distribution of funding; the Levelling Up paper reports the need for investment in culture and heritage outside London, and within poorer areas [38].

For peer review only

The finding that respondents were more likely to have visited a heritage site in the past year, if they were exposed to local heritage, is comparable to previous evidence on spatial access and attendance at museums or galleries in London [18]. Brook et al. (2016) found that attendance rate variation could not be explained solely by people's individual characteristics and better spatial access was an important contributory factor. Authors concluded that well-resourced locales could provide significant "opportunity structures" for cultural engagement [18]. Certainly better spatial access to other types of environmental features is associated with higher usage, such as sports facilities [39], and green space [40]. We found that those with a higher level of historic parks/gardens locally were more likely to have visited this type of green space in the past year. We cannot say why greater exposure increased the likelihood of heritage visits more generally, or whether local exposure equates to higher local site usage in particular. However, it is feasible that nearby heritage could be a significant 'pull factor' as reduced time and effort may be needed for visits, or visibility of local heritage may contribute to social norms and increase interest in visits, whether near or far.

In terms of a main effect, we found no association between heritage exposure and psychological distress. Associations between heritage engagement and better mental health and wellbeing are established [5-7 9 13 17], however, few studies focus on the mental health benefits of spatial exposure to heritage specifically. The lack of association, in our study, could be attributed to use of proximity as a 'proxy' for engagement with local heritage, while, in reality, location of visits was unknown. Future surveys could gather data on how far people travel to engage with heritage. We acknowledge that proximity does not indicate use, or awareness of, local heritage. Residents may lack awareness of local neighbourhood features, such as green space [41] or general amenities [42], and this could be true of local heritage. We did find an interaction effect, amongst those with heritage locally; visitors to heritage showed lower psychological distress. A European-based study looked at associations between neighbourhood green/blue space and mental health [43], and found that although people in highly green/coastal areas experienced better mental health, this relationship did not remain when controlling for recreational visits to these areas. Visit frequency appeared to mediate associations between green/blue space and wellbeing. Authors reasoned that better mental health amongst those in the greenest/bluest areas could be a result of such environmental qualities encouraging visits. Similarly, we found that a combination of proximal heritage, and visits to heritage, benefitted mental health; better spatial exposure appeared to provide a pathway to encourage visits. Our findings contribute to discussions about which type of neighbourhood exposure is key in associations with mental health. Many neighbourhood effects' studies define 'exposure' as 'residential proximity', e.g. green space [44]. It is valuable to examine both spatial exposure, and direct contact/engagement, when considering mental health. The

For peer review only

beneficial association between greenspace and mental health has been well researched, with theories of benefit based on visualising the structure of greenspace, its colours and its landscapes, to engage the human brain, and work to reduce stress, restore attention, and enhance wellbeing [45]. Less is known about how ‘bricks and mortar’ heritage influences mental health. We found that, regardless of green space exposure, those with built heritage in their local area, who had visited heritage, showed lower distress. Historic buildings are considered to improve neighbourhood quality with older buildings creating feelings of grandeur and permanence [35], and exposure to urban environments with historic features, has been perceived more restorative, attractive and interesting than those without [46 47]. Viewing heritage appears to engage the senses, and places considered ‘special’ may stimulate the brain cells that manage emotion [48]. Residents who live in areas with higher proportions of listed buildings, or who regard a building as ‘special’ within their local area, have a greater ‘sense of place’ [10] which, in turn, can be associated with better mental health [49]. Regarding local heritage as ‘special’ could thus be part of the pathway connecting heritage to wellbeing.

Strengths and limitations

Our research exhibited a number of strengths. We used a sample of adults from across the whole of England, weighted to ensure representativeness to the wider population. We explored associations between exposure to heritage, and to visits, and to mental health, within relatively small areas across the whole of England, while other papers did not incorporate spatial data or nation-wide analyses. We explored whether mental health benefits of ’built’ heritage remained regardless of the presence of green space in the local neighbourhood, providing a unique strand to this research. Regarding limitations, this study was cross-sectional; we cannot assume that key variables demonstrate causal associations, and that findings are generalisable within other contexts. We acknowledge that use of LSOA-level analysis may lead to statistical bias from using arbitrarily classified units to report spatial patterning [50]. Our study explores spatial exposure to heritage only, and does not incorporate other factors, which may promote or inhibit accessibility, such as financial access, e.g. admittance fees. Although heritage may often be viewed without admittance. We acknowledge the potential for missing HAR data; lack of resources may result in systematic surveys of different HAR types being undertaken at different times [51]. Additionally, we acknowledge that depressed individuals may be less likely to visit heritage due to mental ill health reducing their drive to engage with heritage.

Policy implications

For peer review only

Our findings of inequities in heritage exposure, as a barrier to heritage engagement, are directly relevant to the UK Government's levelling up agenda. The bill recommends ensuring protection to existing heritage, tackling disparities in heritage access, and improving cultural investment outside London [38]. Going forward the levelling up agenda must address inequalities in heritage exposure as part of a pathway to improve societal mental health. Methods to increase exposure could include the development within areas with fewer heritage sites, of better options for public transport, e.g. subsidised transport to areas with better exposure [33]. Exposure could be improved via investment in heritage in need of reinvigoration, such as at risk sites. Schemes which encourage volunteering to improve at risk assets provide various social, psychological and health benefits to volunteers [52]. Increasing heritage engagement opportunities must consider both geographical and socio-cultural factors [18]. Policy objectives to promote heritage engagement across all cultural groups should seek to redress potentially lower levels of interest among specific groups, such as poorer households [17]. Heritage organisations could expand 'public outreach' activities to neighbourhoods and communities with lower exposure [33], or with lower levels of heritage awareness, and compensate for fewer heritage assets in deprived areas, through increased awareness of what *is* there.

Conclusion

We demonstrated that more income-deprived neighbourhoods in England had lower levels of heritage exposure. Exposure to local heritage increased the likelihood of visiting heritage in general, and exposure to any heritage, or built heritage specifically, was positively associated with mental health, but only amongst those who had visited heritage in the past year. Our findings indicate that both spatial exposure and engaging with heritage through visiting are key. There is much to be gained from improving the structure of exposure. Formulation of strategies for schemes to tackle inequality in exposure in poorer neighbourhoods is necessary. Schemes could include working with communities and areas to improve access, knowledge, and awareness of heritage, and providing investment in 'at risk' heritage and currently non-accessible sites. Such schemes could promote local heritage as 'special', to improve heritage engagement, and ultimately provide benefits to mental health and well-being.

For peer review only

Acknowledgements: We wish to thank Neil Guiden (Data & Analysis Manager) at Historic England for supplying spatial data. We wish to acknowledge the UKHLS participants for their involvement in the study and the UK Data Service for supplying all social survey data used.

Authors contributors: LMac, EG, LM, and RM proposed the research questions. LMac led the project, created GIS variables, and drafted all versions of the article. NN undertook statistical analysis and interpretation. All authors critically revised the article, read and approved final draft.

Funding: LMac, NN and RM are funded by the UK Medical Research Council (MRC) Places and Health Programme (MC_UU_00022/4) and the Chief Scientist Office (CSO) (SPHSU2019) at the MRC/CSO Social and Public Health Sciences Unit, University of Glasgow, UK.

Competing interests: The authors declare no conflict of interest.

Patient consent for publication: Not required.

Ethics approval: The University of Essex Ethics Committee has approved all data collection on Understanding Society. The UKHLS main study gained consent for all data linkages (except to health records) and respondents aged 16 or over provided informed and written consent to participate.

Provenance and peer review: Not commissioned; externally peer reviewed.

Data availability statement: Proposals for collaborative projects and data sharing are welcome. The data presented in this study are available upon reasonable request from the corresponding author.

Supplemental material: This content has been supplied by the authors.

Open access: This is an open access article distributed in accordance with the Creative Commons Attribution 4.0 Unported (CC BY 4.0) license, which permits others to copy, redistribute, remix, transform and build upon this work for any purpose, provided the original work is properly cited, a link to the licence is given, and indication of whether changes were made. See: https://creativecommons.org/licenses/by/ 4.0/.

ORCID: Laura Macdonald https://orcid.org/0000-0002-0593-8079

For peer review only

References

1. Jimenez MP, DeVille NV, Elliott EG, *et al.* Associations between nature exposure and health: A review of the evidence. *International Journal of Environmental Research and Public Health* 2021;18:4790.
2. Twohig-Bennett C, Jones A. The health benefits of the great outdoors: A systematic review and meta-analysis of greenspace exposure and health outcomes. *Environmental research* 2018;166:628-37.
3. Ward Thompson C, Silveirinha de Oliveira E, Wheeler BW*, et al.* Urban Green Spaces and Health, A Review of Evidence. Copenhagen: World Health Organisation, 2016.
4. Historic England. About The List. 2022. https://historicengland.org.uk/listing/the-list/about-the-list/ [Accessed 27 June 2022].
5. Brown JL, MacDonald R, Mitchell R. Are People Who Participate in Cultural Activities More Satisfied with Life? *Social Indicators Research* 2015;122:135-46.
6. Fujiwara D, Cornwall T, Dolan P. Heritage and Wellbeing. London: English Heritage, 2014.
7. Leadbetter C, O'Connor N. Healthy Attendance? The Impact of Cultural Engagement and Sports Participation on Health and Satisfaction with Life in Scotland. Edinburgh: Scottish Government, 2013.
8. Lakey J, Smith N, Oskala A*, et al.* Culture, sport and well-being. Findings from the Understanding Society Youth Survey. London: NatCen Social Research, 2017.
9. Mak HW, Coulter R, Fancourt D. Associations between community cultural engagement and life satisfaction, mental distress and mental health functioning using data from the UK Household Longitudinal Study (UKHLS): are associations moderated by area deprivation? *BMJ open* 2021;11:e045512.
10. Bradley DP, Coombes M, Tranos E*, et al.* Sense of place and social capital and the historic built environment: Report of research for English Heritage. Newcastle: Newcastle University and Bradley Research and Consulting, 2009.
11. Harris PB, Werner CM, Brown BB*, et al.* Relocation and privacy regulation: A cross-cultural analysis. *Journal of environmental psychology* 1995;15:311-20.
12. Rollero C, De Piccoli N. Does place attachment affect social well-being? *European Review of Applied Psychology* 2010;60:233-38.
13. Darvill T, Heaslip V, Barrass K. Heritage and well-being: Therapeutic places past and present. In: Darvill T, Barrass K, Drysdale L, et al., eds. Historic Landscapes and Mental Well-being. Oxford: Archaeopress, 2019:17.
14. Grossi E, Tavano Blessi G, Sacco PL. Magic moments: determinants of stress relief and subjective wellbeing from visiting a cultural heritage site. *Culture, Medicine, and Psychiatry* 2019;43:4-24.
15. Sofaer J, Davenport B, Sørensen MLS*, et al.* Heritage sites, value and wellbeing: learning from the COVID-19 pandemic in England. *International Journal of Heritage Studies* 2021;27:1117-32.
16. National Statistics. Taking Part 2019/20 - Heritage. 2020. https://www.gov.uk/government/statistics/taking-part-201920-heritage [Accessed 08 February 2022].
17. Lakey J, Smith N, Oskala A*, et al.* Culture, sport and well-being. Findings from the Understanding Society Adult Survey. London: NatCen Social Research, 2017.
18. Brook O. Spatial equity and cultural participation: how access influences attendance at museums and galleries in London. *Cultural Trends* 2016;25:21-34.
19. OCSI. 'Left behind' Neighbourhoods: Community data dive. 2021. https://www.appg-leftbehindneighbourhoods.org.uk/wp-content/uploads/2021/06/APPG-Community-Data-Dive-Report-for-APPG-S7.pdf [Accessed 09 February 2022].
20. HM Government. Levelling Up the United Kingdon. London: Her Majesty's Stationery Office, 2022.

For peer review only

21. Office for National Statistics. Census geography. 2021. https://www.ons.gov.uk/methodology/geography/ukgeographies/censusgeography [Accessed 09 May 2022].
22. UK Government. English indices of deprivation 2015. 2015. https://www.gov.uk/government/statistics/english-indices-of-deprivation-2015 [Accessed 20 October 2021].
23. Office for National Statistics. Lower layer Super Output Area population estimates. 2021. https://www.ons.gov.uk/peoplepopulationandcommunity/populationandmigration/populationestimates/datasets/lowersuperoutputareamidyearpopulationestimates [Accessed 20 October 2021].
24. Historic England. What is the Heritage at Risk Programme? 2022. https://historicengland.org.uk/advice/heritage-at-risk/types/ [Accessed 21 March 2022].
25. Ordnance Survey. OS Open Greenspace. 2021. https://www.ordnancesurvey.co.uk/business-government/products/open-map-greenspace.
26. Office for National Statistics. Lower Layer Super Output Area (LSOA) boundaries. 2016. https://data.gov.uk/dataset/fa883558-22fb-4a1a-8529-cffdee47d500/lower-layer-super-output-area-lsoa-boundaries.
27. Goldberg DP. User's guide to the General Health Questionnaire. Windsor, 1988.
28. Astell-Burt T, Mitchell R, Hartig T. The association between green space and mental health varies across the lifecourse. A longitudinal study. *Journal of Epidemiology and Community Health* 2014;68:578-83.
29. Mak HW, Coulter R, Fancourt D. Associations between neighbourhood deprivation and engagement in arts, culture and heritage: evidence from two nationally-representative samples. *BMC public health* 2021;21:1-10.
30. Von Elm E, Altman DG, Egger M*, et al.* The Strengthening the Reporting of Observational Studies in Epidemiology (STROBE) statement: guidelines for reporting observational studies. *Annals of internal medicine* 2007;147:573-77.
31. StataCorp. Stata Statistical Software. College Station, Texas: StataCorp LLC, 2021.
32. Department for Transport. Travel by vehicle availability, income, ethnic group, household type, mobility status and NS-SEC. 2020. https://www.gov.uk/government/statistical-data-sets/nts07-car-ownership-and-access#full-publication-update-history [Accessed 31 May 2022].
33. Moldavanova AV, Meloche L, Thompson TL. Understanding the geography of access to cultural amenities: The case of Metropolitan Detroit. *Journal of Urban Affairs* 2021:1-26.
34. Ahlfeldt GM, Holman N, Wendland N. An assessment of the effects of conservation areas on value. London: London School of Economics and Political Science, 2012.
35. Maeer G, Robinson A, Hobson M. Values and benefits of heritage A research review. London: Heritage Lottery Fund, 2016.
36. Braubach M, Fairburn J. Social inequities in environmental risks associated with housing and residential location—a review of evidence. *European journal of public health* 2010;20:36-42.
37. Macintyre S, Macdonald L, Ellaway A. Do poorer people have poorer access to local resources and facilities? The distribution of local resources by area deprivation in Glasgow, Scotland. *Social science & medicine* 2008;67:900-14.
38. The Heritage Alliance. Levelling Up White Paper Summary for Heritage. London: The Heritage Alliance, 2022.
39. Giles-Corti B, Donovan RJ. The relative influence of individual, social and physical environment determinants of physical activity. *Social Science & Medicine* 2002;54:1793–812.
40. Pyky R, Neuvonen M, Kangas K*, et al.* Individual and environmental factors associated with green exercise in urban and suburban areas. *Health & place* 2019;55:20-28.

For peer review only

41. Macintyre S, Macdonald L, Ellaway A. Lack of agreement between measured and self-reported distance from public green parks in Glasgow, Scotland. *International Journal of Behavioral Nutrition and Physical Activity* 2008;5:1-8.
42. Macdonald L, Kearns A, Ellaway A. Do residents' perceptions of being well-placed and objective presence of local amenities match? A case study in West Central Scotland, UK. *BMC Public Health* 2013;13:1-11.
43. White MP, Elliott LR, Grellier J*, et al.* Associations between green/blue spaces and mental health across 18 countries. *Scientific reports* 2021;11:1-12.
44. Bratman GN, Hamilton JP, Daily GC. The impacts of nature experience on human cognitive function and mental health. *Annals of the New York Academy of Sciences* 2012;1249:118–36.
45. Markevych I, Schoierer J, Hartig T*, et al.* Exploring pathways linking greenspace to health: Theoretical and methodological guidance. *Environmental research* 2017;158:301-17.
46. Bornioli A, Parkhurst G, Morgan PL. Psychological wellbeing benefits of simulated exposure to five urban settings: an experimental study from the pedestrian's perspective. *Journal of transport & health* 2018;9:105-16.
47. Bornioli A, Parkhurst G, Morgan PL. The psychological wellbeing benefits of place engagement during walking in urban environments: A qualitative photo-elicitation study. *Health & place* 2018;53:228-36.
48. National Trust. Why Places Matter to People Research Report. Wiltshire: National Trust, 2018.
49. Williams A, Kitchen P. Sense of Place and Health in Hamilton, Ontario: A Case Study. *Social indicators research* 2012;108:257-76.
50. Openshaw S. The Modifiable Areal Unit Problem. Norwich, England: Geobooks, 1984.
51. Historic England. Heritage at Risk Statistics: Method Statement. London: Historic England, 2021.
52. Lewis C, Siriwardena N, Laparidou D*, et al.* Wellbeing in Volunteers on Heritage at Risk Projects. London: Historic England, 2021.

For peer review only

Supplemental table 1. EIMD Income Quintile descriptive – population, geographical area, heritage.

EIMD Income	Population (mean)	Area km² (mean)	Listed buildings	Historic Parks/ gardens	Scheduled Monuments	All sites
**1 (most deprived)**	11075909 (1686)	3428 (0.52)	19880	203	277	20360
**2**	11149857 (1697)	7547 (1.15)	41189	292	913	42394
**3**	11016312 (1677)	26114 (3.98)	75084	470	4170	79724
**4**	10820663 (1647)	50252 (7.65)	125462	790	7904	134156
**5 (least deprived)**	10723586 (1632)	43089 (6.56)	113923	927	5893	120743
**Total**	54786327 (1668)	130432 (3.97)	375538	2682	19157	397377

Supplemental table 2. Sample descriptive (percentage by each category)

**Age**	<20 years old	5.7
	20-29 years old	12.8
	30-39 years old	15.1
	40-49 years old	17.7
	50-59 years old	16.9
	60-69 years old	15.4
	70+ years old	16.2
	Base	30431
**Sex**	Male	46.5
	Females	53.5
	Base	30431
**EIMD income**	Q1 (most deprived)	18.1
	Q2	19.2
	Q3	21.3
	Q4	21.7
	Q5 (least deprived)	19.8
	Base	30431
**Ethnicity**	White	91.2

For peer review only

	Other	8.8
	Base	30420
**Education**	Degree or higher	35.4
	GCSEs/A-levels	41.7
	Other qualification	10.1
	No qualification	12.8
	Base	30377
**Job type (NS-SEC)**	Managerial, admin & professional	24.4
	Intermediate occupations	13.5
	Routine & manual occupations	20.0
	Not in paid employment	42.1
	Base	30089
**Housing tenure**	Owner occupier	70.1
	Social renter	17.7
	Private renter	12.2
	Base	29932
**Long-term illness/mobility**	No long-term illness or mobility issue	52.3
	Long-term illness	15.2
	Long-term illness and mobility issue	32.5
	Base	30420
**Marital/cohabitating status**	Single	23.1
	Married/cohabiting	61.8
	Divorced/separated	8.4
	Widowed	6.7
	Base	30361

For peer review only

Reporting checklist for cross sectional study.

Based on the STROBE cross sectional guidelines.

Instructions to authors

Complete this checklist by entering the page numbers from your manuscript where readers will find each of the items listed below.

Your article may not currently address all the items on the checklist. Please modify your text to include the missing information. If you are certain that an item does not apply, please write "n/a" and provide a short explanation.

Upload your completed checklist as an extra file when you submit to a journal.

In your methods section, say that you used the STROBE cross sectionalreporting guidelines, and cite them as:

von Elm E, Altman DG, Egger M, Pocock SJ, Gotzsche PC, Vandenbroucke JP. The Strengthening the Reporting of Observational Studies in Epidemiology (STROBE) Statement: guidelines for reporting observational studies.

For peer review only

		Reporting Item	Page Number
**Title and abstract**			
Title	#1a	Indicate the study’s design with a commonly used term in the title or the abstract	2
Abstract	#1b	Provide in the abstract an informative and balanced summary of what was done and what was found	2

**Introduction**			
Background / rationale	#2	Explain the scientific background and rationale for the investigation being reported	3-5
Objectives	#3	State specific objectives, including any prespecified hypotheses	5
**Methods**			
Study design	#4	Present key elements of study design early in the paper	5-9
Setting	#5	Describe the setting, locations, and relevant dates, including periods of recruitment, exposure, follow-up, and data collection	5-9
Eligibility criteria	#6a	Give the eligibility criteria, and the sources and methods of selection of participants.	8
	#7	Clearly define all outcomes, exposures, predictors, potential confounders, and effect modifiers. Give diagnostic criteria, if applicable	5-9
Data sources / measurement	#8	For each variable of interest give sources of data and details of methods of assessment (measurement). Describe comparability of assessment methods if there is more than one group. Give information separately for for exposed and unexposed groups if applicable.	5-9
Bias	#9	Describe any efforts to address potential sources of bias	5-9

For peer review only

Study size	#10	Explain how the study size was arrived at	5-9
Quantitative variables	#11	Explain how quantitative variables were handled in the analyses. If applicable, describe which groupings were chosen, and why	5-9
Statistical methods	#12a	Describe all statistical methods, including those used to control for confounding	8-9
Statistical methods	#12b	Describe any methods used to examine subgroups and interactions	8-9
Statistical methods	#12c	Explain how missing data were addressed	9
Statistical methods	#12d	If applicable, describe analytical methods taking account of sampling strategy	n/a
Statistical methods	#12e	Describe any sensitivity analyses	n/a
**Results**			
Participants	#13a	Report numbers of individuals at each stage of study—eg numbers potentially eligible, examined for eligibility, confirmed eligible, included in the study, completing follow-up, and analysed. Give information separately for for exposed and unexposed groups if applicable.	n/a
Participants	#13b	Give reasons for non-participation at each stage	n/a
Participants	#13c	Consider use of a flow diagram	n/a

For peer review only

Descriptive data	#14a	Give characteristics of study participants (eg demographic, clinical, social) and information on exposures and potential confounders. Give information separately for exposed and unexposed groups if applicable.	(supplemental table)
Descriptive data	#14b	Indicate number of participants with missing data for each variable of interest	(supplemental table)
Outcome data	#15	Report numbers of outcome events or summary measures. Give information separately for exposed and unexposed groups if applicable.	n/a
Main results	#16a	Give unadjusted estimates and, if applicable, confounder-adjusted estimates and their precision (eg, 95% confidence interval). Make clear which confounders were adjusted for and why they were included	9-12
Main results	#16b	Report category boundaries when continuous variables were categorized	7
Main results	#16c	If relevant, consider translating estimates of relative risk into absolute risk for a meaningful time period	n/a
Other analyses	#17	Report other analyses done—e.g., analyses of subgroups and interactions, and sensitivity analyses	9-10

Discussion

For peer review only

Key results	#18	Summarise key results with reference to study objectives	13
Limitations	#19	Discuss limitations of the study, taking into account sources of potential bias or imprecision. Discuss both direction and magnitude of any potential bias.	15
Interpretation	#20	Give a cautious overall interpretation considering objectives, limitations, multiplicity of analyses, results from similar studies, and other relevant evidence.	13-16
Generalisability	#21	Discuss the generalisability (external validity) of the study results	15
**Other Information**			
Funding	#22	Give the source of funding and the role of the funders for the present study and, if applicable, for the original study on which the present article is based	17

Notes:

- 14a: 15 (supplementary table)
- 14b: 15 (supplementary table)
- 16a: 9-10 (and tables) The STROBE checklist is distributed under the terms of the Creative Commons Attribution License CC-BY. This checklist was completed on 26. July 2022 using https://www.goodreports.org/, a tool made by the EQUATOR Network in collaboration with Penelope.ai

For peer review only

BMJ Open

Is spatial exposure to heritage associated with visits to heritage and to mental health? A cross-sectional study using data from the UK Household Longitudinal Study (UKHLS).

Journal:	*BMJ Open*
Manuscript ID	bmjopen-2022-066986.R1
Article Type:	Original research
Date Submitted by the Author:	23-Jan-2023
Complete List of Authors:	Macdonald, Laura; University of Glasgow MRC/CSO Social and Public Health Sciences Unit, MRC Social and Public Health Sciences Unit; University of Glasgow MRC/CSO Social and Public Health Sciences Unit,<br>Nicholls, Natalie; University of Glasgow MRC/CSO Social and Public Health Sciences Unit<br>Gallou, Eirini; Historic England, Policy and Evidence<br>Monckton, Linda; Historic England, Policy and Evidence<br>Mitchell, Richard; University of Glasgow MRC/CSO Social and Public Health Sciences Unit
**Primary Subject Heading**:	Public health
Secondary Subject Heading:	Mental health
Keywords:	MENTAL HEALTH, STATISTICS & RESEARCH METHODS, PUBLIC HEALTH

SCHOLARONE™
Manuscripts

I, the Submitting Author has the right to grant and does grant on behalf of all authors of the Work (as defined in the below author licence), an exclusive licence and/or a non-exclusive licence for contributions from authors who are: i) UK Crown employees; ii) where BMJ has agreed a CC-BY licence shall apply, and/or iii) in accordance with the terms applicable for US Federal Government officers or employees acting as part of their official duties; on a worldwide, perpetual, irrevocable, royalty-free basis to BMJ Publishing Group Ltd ("BMJ") its licensees and where the relevant Journal is co-owned by BMJ to the co-owners of the Journal, to publish the Work in this journal and any other BMJ products and to exploit all rights, as set out in our licence.

The Submitting Author accepts and understands that any supply made under these terms is made by BMJ to the Submitting Author unless you are acting as an employee on behalf of your employer or a postgraduate student of an affiliated institution which is paying any applicable article publishing charge ("APC") for Open Access articles. Where the Submitting Author wishes to make the Work available on an Open Access basis (and intends to pay the relevant APC), the terms of reuse of such Open Access shall be governed by a Creative Commons licence – details of these licences and which Creative Commons licence will apply to this Work are set out in our licence referred to above.

Other than as permitted in any relevant BMJ Author's Self Archiving Policies, I confirm this Work has not been accepted for publication elsewhere, is not being considered for publication elsewhere and does not duplicate material already published. I confirm all authors consent to publication of this Work and authorise the granting of this licence.

For peer review only

Is spatial exposure to heritage associated with visits to heritage and to mental health? A cross-sectional study using data from the UK Household Longitudinal Study (UKHLS).

Laura Macdonald [a], Natalie Nicholls [a], Eirini Gallou [b], Linda Monckton [b], Richard Mitchell [a].

a. MRC/CSO Social and Public Health Sciences Unit, University of Glasgow, Glasgow, UK

b. Policy and Evidence, Historic England, London, UK

Correspondence to:

Mrs Laura Macdonald,

Places and Health Programme,

MRC/CSO Social and Public Health Sciences Unit,

University of Glasgow.

laura.macdonald@glasgow.ac.uk

For peer review only

Abstract

Objectives: Existing research highlights the beneficial nature of heritage engagement for mental health, but engagement varies geographically and socially, and few studies explore spatial exposure (i.e., geographic availability) to heritage and heritage visits. Our research questions were 'does spatial exposure to heritage vary by area income deprivation?', 'is spatial exposure to heritage linked to visiting heritage?', and 'is spatial exposure to heritage linked to mental health?'. Additionally, we explored whether local heritage is associated with mental health regardless of the presence of green space.

Design: Data collected January 2014-June 2015 via the UK Household Longitudinal Study (UKHLS) wave 5. Our study is cross-sectional.

Setting: UKHLS data was either collected via face-to-face interview or on-line questionnaire.

Participants: 30,431 adults (16 years+) (13,676 males, 16,755 females). Participants geocoded to Lower Super Output Area (LSOA) 'neighbourhood' and 'English Index Multiple Deprivation' (EIMD) 2015 Income score.

Main exposures/outcome measures: LSOA-level heritage exposure and green space exposure (i.e., population and area densities); heritage site visit in past year (outcome, binary: no, yes); mental distress (outcome, General Health Questionnaire-12, binary: less distressed 0-3, more distressed 4+).

Results: Heritage varied by deprivation, the most deprived areas (Income Quintile (Q)1:1.8) had fewer sites per 1000 population than the least deprived (Q5:11.1) (p<0.01). Compared to those with no LSOA-level heritage, those with heritage exposure were more likely to have visited a heritage site in the past year (OR: 1.12 (95%CI: 1.03-1.22)) (p<0.01). Amongst those with heritage exposure, visitors to heritage had a lower predicted probability of distress (0.171 (95%CI: 0.162-0.179)) than non-visitors (0.238 (95%CI: 0.225-0.252)) (p<0.001).

Conclusions: Our research contributes to evidence for the wellbeing benefits of heritage and is highly relevant to the government's levelling-up heritage strategy. Our findings can feed into schemes to tackle inequality in heritage exposure to improve both heritage engagement and mental health.

Keywords: heritage, GIS, inequalities, mental health.

For peer review only

Strengths and limitations of this study

- Our paper is the first to explore associations between spatial exposure to heritage, and visits to heritage, and mental health, within relatively small areas across England.
- We use a sample of adults from across England, with sampling weighted to ensure representativeness to the wider population of England.
- We use a variety of LSOA-level measures to capture exposure to heritage, i.e. population and area densities, and percentage of sites as 'at risk'.
- We explore whether associations between mental health and heritage remained regardless of the presence of local green space, providing a unique strand to this research.
- Our study may be limited in that we do not know whether UKHLS respondents visit heritage sites within their local area or further afield.

Introduction

Population health research has paid much attention to the health benefits of exposure to natural environments [1]. Regular contact with green space has been associated with better physical health, e.g. through reduced risk of hypertension, stroke, cardiovascular disease and asthma, and improved immune function [2]. There is stronger evidence for mental health benefits of time spent in green and natural space, such as improved cognitive function and brain activity [1], greater social cohesion, and sense of community, relaxation and restorative psychological effects, and lower levels of depression, anxiety and stress [3].

A much smaller evidence base suggests that visiting heritage may also benefit mental health and wellbeing. In this study, we define heritage as a building, monument, site or area classified as having 'a degree of significance' worthy of consideration in planning related decisions, due to its architectural or historic interest [4]. Heritage assets may be 'designated' by the Secretary of State for Digital, Culture, Media and Sport (DCMS) as advised by Historic England, or recognised for their significance by local planning authorities, while 'World heritage sites' are further recognised for their international value by bodies such as UNESCO. People who frequently visit heritage sites report higher life satisfaction [5-7], enhanced levels of self-esteem and happiness [8], and lower levels of mental distress [9]. Residents of places with greater numbers of heritage sites have a stronger 'sense of place' [10] and higher levels of place attachment have been associated with better quality of life [11], and 'social wellbeing' [12]). Studies have also shown that engaging with heritage can be psychologically restorative [13], and that visits to cultural heritage sites can reduce stress [14]. The

For peer review only

denial of access to heritage during the Covid-19 lockdowns also provided evidence for its benefits. Awareness of the value of heritage to mental wellbeing was reportedly reinforced by being unable to access it [15].

One problem with studies of both green space and heritage is that they do not often allow for each other, or acknowledge the overlap between them. Historic parks and gardens are 'cultural assets' in that they are commonly designed or created by and for people with a purpose. They are often cultural landscapes in their own right, but many parks and gardens contain historic buildings or have historic value, and many monuments and buildings are close to or surrounded by green spaces. Furthermore, the parts of towns and cities that contain more (or less) heritage might well also contain more (or less) green space and trying to 'untangle' the contributions of each environment to health and wellbeing is a difficult task. Overall, though, evidence suggests that exposure to heritage has the potential to benefit health, and in particular, mental health.

Despite the potential benefits, visits to heritage are unequal. People living in more deprived areas are less likely to engage in the arts, culture and heritage regardless of personal socio-economic status [9]. In the UK in 2019/2020, a higher proportion of adults resident in the least deprived areas (83%) had visited sites in the past year than those resident in the most deprived (50%) [16]. Heritage engagement levels vary by individual characteristics too with, e.g., those in employment, and in higher socio-economic groups, more likely to visit [17]. Geographical availability of cultural assets, such as museums and libraries is associated with visits or engagement [18], but availability is unequal across the UK with less deprived areas showing better access [19]. In this study, we refer to this geographical availability factor as 'exposure.' There is surprisingly little evidence about exposure to heritage, about how it interacts with the socio-economic characteristics of the population and whether it is implicated in any health benefits. Such evidence is important to both agencies concerned with improving and equalising population health, and those seeking to understand the value and potential of heritage. If levels of exposure make a difference for both engagement and health, there is potential for place-based schemes to improve and equalise it.

To address this gap, in this paper we explore whether spatial exposure to designated heritage (i.e. listed buildings, scheduled monuments and historic parks/gardens) varies by area income deprivation within England, and whether spatial exposure to heritage is linked to visits to heritage (unadjusted and adjusted for socio-demographic factors). We then explore whether exposure to heritage is associated with mental health among all residents, and those who are visitors and non-visitors (controlling for socio-demographic factors and other green space). Additionally, we explore 'built' heritage, specifically, to understand whether exposure to listed buildings and scheduled

For peer review only

monuments is associated with mental health benefits beyond those associated with exposure to green space. We consider whether the physical presence of heritage in an area is related to psychological wellbeing among those living in proximity to it and disentangle the potential overlap in associations with well-being related to heritage exposure and to green space exposure. Our research is timely, with, in the 2022 Levelling Up paper, the UK government showing a commitment to tackling socio-spatial inequalities in heritage access and engagement [20].

In summary, our three research questions are: 'does spatial exposure to heritage (and heritage 'at risk') vary by area deprivation?', 'Is spatial exposure to heritage linked to visiting heritage?', and 'Is spatial exposure to heritage (i.e., any, or 'built' heritage specifically) linked to mental health among heritage visitors and non-visitors?'.

Methods

To answer our research questions, we needed four data components describing: (i) local area population, context and socio-economic situation; (ii) spatial exposure to heritage; (iii) spatial exposure to other green spaces; and (iv) individual level visits to heritage, health, and socio-demographic covariates. These data needed a common spatial framework to represent, and georeference individuals (i.e., residential location related to a ground system of geographic coordinates) to, the 'local area' or 'neighbourhood'. The spatial framework was Lower Super Output Area (LSOA) (as individual addresses or postal codes not available). LSOAs are small area units created for collecting and reporting statistics about the UK population [21]. They are socially homogeneous and consistent in population size; however, their geographical size varies according to level of urban/rural (e.g., rural LSOAs are geographically larger as their populations are more dispersed). LSOAs have a median area size of 0.46 km^2 and median population size of 1,500 residents or 650 households (see supplemental table 1 for more information). There are 32,844 LSOAs in England and they are commonly used as a proxy for 'neighbourhood'.

Data

(i) local area population, context and socio-economic situation

We obtained the English Index of Multiple Deprivation (EIMD) 2015 (income domain) ranks and quintiles [22] and 2015 mid-year population estimates [23] to describe LSOA population and socio-economic situation. We did not use the full EIMD as it includes barriers to local services and health variables, therefore, there may have been some circularity in investigating whether the full EIMD was associated with access to heritage/green space and/or mental health. An EIMD Income quintile

For peer review only

descriptive is included within supplemental table 1 (including total and mean population/size per quintile).

(ii) and (iii) Spatial exposure to heritage and other green spaces.

Heritage data: We obtained spatial data on all nationally protected historic buildings and sites from Historic England for 2014 [4]. The National Heritage List for England originated in 1882 when the first powers of protection were established and became a statutory 'Listing' after World War Two (mid-1940s). Currently, the List holds over 400,000 entries and draws together all scheduled monuments, listed buildings, registered landscapes, battlefields, and protected wrecks. Historic England continuously updates the list, curated from the DCMS [4]. In our analysis we included listed buildings (points only – in compliance with Ordnance Survey (OS) licence), scheduled monuments (polygons), and historic parks/gardens (polygons). We excluded wreck-sites (not visible from land) and excluded battlefields as small numbers of these sites (n=47, sited within 0.1% of LSOAs only) would not allow meaningful comparison across income quintiles. Listed buildings are buildings (e.g., residences, farmhouses, churches etc.) recognised as being of special architectural or historic interest. Scheduled Monuments are historic sites of national importance that are legally protected, such as, Roman remains, burial mounds, castles, bridges, earthworks, etc. Historic parks/gardens are protected 'designed' landscapes, including a range of planned open spaces, such as public parks, cemeteries, private house grounds etc. The dataset also distinguished 'Heritage at Risk' (HAR) sites. HAR is an official description of a heritage asset added to the HAR register that is maintained by Historic England, and records the condition of designated heritage assets after assessment. Such sites are vulnerable to loss due to "neglect, decay or inappropriate development" and are in need of safeguarding and protection [24].

Other green space data: We obtained data on other green space for England from the OS Open Greenspace database. Data for 2014 were not available, with the closest temporal match being July 2017 [25]. These were vector data (scale 1:25000) including polygon boundaries of green spaces. We selected green spaces that we considered 'natural heritage', including: allotments and community growing spaces; cemeteries and other religious grounds; golf courses; and parks/gardens. We excluded spaces associated with sport/play: tennis courts; bowling greens; other sports facilities; playing fields; and play spaces. Golf courses may be regarded as sports facilities; however, these were included as they are often geographically large and highly visual green spaces, which may contain publicly accessible footpaths and can be surrounded by other green space or coastal walks (i.e., proximal access is available). Some heritage sites included in the data from Historic England were likely to also feature in the OS data. To ensure we did not double count these, we overlaid the

For peer review only

two datasets in a Geographic Information System (GIS), ArcMap 10.8.1, to check for and remove duplicates; any feature captured in the data from Historic England was removed from the OS green space data. The remaining features are referred to as 'other green space'. We obtained LSOA boundaries (2011) [26] and used the 'Overlay' 'Spatial Join' tool to link the points (or polygon centroids/centre points) in the heritage and other green spaces datasets to LSOA boundaries. If a site (polygon) was spread across more than one LSOA, it was linked to the LSOA in which its centroid was located. We then calculated a count of each heritage feature and a count of each other green space feature in each LSOA.

Exposure variables: We then used IBM SPSS statistics v28 to calculate three categorical exposure measures of heritage (at LSOA level). These were 'presence or absence of heritage sites', 'heritage sites per 1000 population', and 'heritage sites per kilometre squared (km^2)'. Using these three measures allowed us to model whether certain 'levels of' heritage were important, and allowed us to account for variation in the geographical size of LSOAs. The per population and per area exposure variables were grouped into 'none', 'low-medium levels (1-4 sites)', and 'higher levels' (five or more sites)'. Due to no existing methodology on appropriate groupings of exposure we based our thresholds on the distribution of values and on percentile representations i.e., numbers roughly represented none, 25th-50th percentile, and 75th percentile, respectively. We also used the same method detailed above to create the per population and per area exposure variables for: 'built' heritage (i.e., listed buildings/scheduled monuments), historic parks/gardens, other green space (non-heritage), and any green space (heritage and non-heritage combined). We joined the area measures of heritage, 'built' heritage, other green spaces, and any green spaces to individual level data via the individual LSOA of residence. UKHLS individuals' LSOA codes were accessed via a UK Data Service special licence.

(iv) individual-level visits to heritage, health, and socio-demographic covariates

Visits to heritage: we undertook a secondary analysis with individual-level data drawn from wave five (2014) of the UK Household Longitudinal Study (UKHLS, 2021) (see supplemental table 2 for sample descriptive). Data were obtained via the UK Data Service. The UKHLS is a large panel survey, which used a clustered and stratified, probability sampling method, and tracked around 31,000 people in England since 2009 (for full details on the survey and its methodology including sampling methods see [27]).We used wave five as it was the most recent wave with questions about visits to heritage. Respondents were asked how often (i.e., 'not once in the last 12 months', 'once in the last 12 months', 'twice in the last 12 months', 'at least 3 or 4 times a year', 'at least once a month', 'at least once a week') they visited each of these heritage sites:-'a city or town with historic character',

For peer review only

‘a historic building’, ‘a historic place of worship’, ‘a historic park or garden, ‘a place of industrial history or historic transport system’, ‘a monument such as a castle, fort or ruin’, ‘a site of archaeological interest’ or a ‘sports heritage site’, in the past year. We did not know whether visits to heritage were in respondent’s LSOA or not.

Mental health measure and covariates: UKHLS carries a General Health Questionnaire (GHQ-12) subjective wellbeing measure, which we included as a measure of mental health. The GHQ-12 includes 12 questions evaluating general mental health functioning and distress. Responses use a 4-point scoring system, i.e. better than normal, same as usual, worse than usual, or much worse than usual, and responses are summed to a score varying from 0 to 12 [28]. We modelled the score as a binary variable lower distress (0-3) and higher distress (i.e., minor psychiatric morbidity)’ (4+); the range of GHQ-12 values was not appropriate for use as a continuous numeric outcome as it is a bounded interval scale, with values being of an integer format. GHQ has been clinically validated, is a screening tool, and the threshold of four deemed valid to represent minor psychiatric morbidity ‘case-ness’ for the UK population, as this low level protects sensitivity[29]. Additionally, it has been used previously in green space and mental health research [30]).Covariates were chosen based on their established associations with visits to heritage and/or mental health [17 31], and examination of full model coefficients confirmed the appropriateness of inclusion of these variables within models. They were sex; age-group (10-year intervals); EIMD income (quintiles); household composition (‘single adult’, ‘single adult with children under 16’, ‘two adults or more’, ‘two adults (or more) with children under 16’); ethnicity (‘White’, ‘Black, Asian and Minority Ethnic (BAME)/mixed’ (numbers did not permit more specific groupings)), highest education qualification (‘degree/other higher degree’, ‘A-levels/GCSEs’, ‘other qualification’, ‘no qualification’), socio-economic status (NS-SEC - ‘managerial/ professional’, ‘intermediate/small employment/own account’, ‘lower supervision/lower technician/semi-routine/routine’, ‘not in (paid) employment’), housing tenure (‘owner occupier’, ‘social renter’, ‘private renter’), and long-standing health condition/mobility (‘none’, ‘condition without limited mobility’, ‘condition with limited mobility’).

Patient and public involvement: We did not involve study participants in the development of the research question, design and implementation of the study or interpretation of the results.

STROBE: Strengthening the Reporting of Observational studies in Epidemiology cross-sectional reporting guidelines were adhered to within this study [32].

See figure one for a flow chart summary of GIS and individual data, including final numbers in analyses. **Figure 1. about here**

For peer review only - http://bmjopen.bmj.com/site/about/guidelines.xhtml

Statistical analysis

To explore whether spatial exposure to heritage sites varies by deprivation, we compared mean/median densities of sites (per 1000 population, per square kilometre (km^2)), across area income deprivation quintiles, using the Kruskal Wallis test, as the distribution of these sites indicated that parametric comparisons were not appropriate. We also calculated percentage of heritage sites as 'at risk' across deprivation quintile.

To explore whether spatial exposure to heritage was linked to visiting heritage, we used multilevel logistic regression due to the inclusion of binary outcomes and random effects necessary due to sampling method. Regression was used to determine the association between heritage exposure measures (i.e., presence of sites (none, 1+), sites per population (none, 1-4, 5+) and per area (none, 1-4, 5+)), and whether a respondent had visited a heritage site in the past year (i.e., 'no visits', 'one or more visits yearly')), controlling for individual socio-demographic covariates (listed previously). We undertook further similar modelling to explore whether visits to historic parks/gardens, and scheduled monuments, specifically, were related to exposure to these particular sites respectively.

To explore whether spatial exposure to heritage was linked to mental health, we used multilevel logistic regression to determine the association between heritage exposure measures (as above) and the mental health measure (binary outcome). We controlled for socio-demographic variables, 'visits to heritage' and exposure to 'other green space'. Other green space was included as a confounder to account for potential associations between green space and mental health; such associations were established in prior research [3]. Models included, and tested, a 'heritage exposure/visits' interaction to investigate moderating effects on mental health (any non-significant terms were removed and models re-run with main effects only). Furthermore, we repeated models with 'built' heritage only (i.e., listed buildings and scheduled monuments) and controlled for 'all green space' (i.e., historic parks/gardens and other green space combined).

In all multilevel models, primary sampling unit (PSU) was set as random effect, weighted by the cross-sectional individual weights supplied by the data providers (see [33] for further information on the PSU). Model results are presented as adjusted odds ratios (OR) and, for independent factors with more than two levels, predicted probabilities (of visits, and of poorer mental health). In addition, post hoc multiple comparison testing was applied. Where applicable, to allow interpretation of results of a model's interaction term, predicted probabilities were presented, and subject to post hoc testing. Regarding missing data, a complete case approach was used, i.e., statistical analysis included only respondents for which there was no missing data on the variables of interest (n=30,431). Those included and excluded from analysis did not vary in terms sex and

education, but did somewhat for age, income, and ethnicity, (included/excluded: 53%/54% female, 36%/36% degree or higher, mean age: 48/43 years, 21%/27% in most deprived areas, 82%/76% white). Multilevel models performed in STATA [34], with a global significance set at 5%.

Results

Heritage exposure varied by area deprivation in a manner dependent on the type of exposure (descriptive information on heritage numbers per quintile available within supplementary table 2). Table 1 shows the distribution of heritage sites per population, and per area/km², by EIMD income quintile. The most deprived areas (Q1) had lower numbers of heritage per 1000 population, while Q4 and Q5 had the highest numbers. This was evident for all three types of heritage analysed together and separately. The pattern for the numbers per area across income deprivation was less clear. The most deprived areas (Q1) had the lowest numbers of listed buildings (mean 9.08), scheduled monuments (0.06) and sites combined (9.23) (see table 1). Q2 had the highest number of listed buildings (12.92), and combined sites (13.10), while Q5 had the highest numbers of scheduled monuments (0.11). Though significant, there was very little variation in density of historic parks/gardens per area across the deprivation quintiles.

For peer review only

Table 1. Distribution of heritage sites (2014) per 1000 population, and per area/km², by EIMD Income (2015) quintile.

	Listed buildings Mean/median/ (Interquartile range)		Historic parks and gardens Mean/median/ (Interquartile range)		Scheduled Monuments Mean/median/ (Interquartile range)		Overall Mean/median/ (Interquartile range)	
**EIMD Income**	**per 1000 people**	**per km²**	**per 1000 people**	**per km²**	**per 1000 people**	**per km²**	**per 1000 people**	**per km²**
**1 (most deprived)**	1.79/0.00 (0.00-0.89)	9.08/0.00 (0.00-4.61)	0.02/0.00 (0.00-0.00)	0.09/0.00 (0.00-0.00)	0.03/0.00 (0.00-0.00)	0.06/0.00 (0.00-0.00)	1.83/0.00 (0.00-0.99)	9.23/0.00 (0.00-4.80)
**2**	3.73/0.48 (0.00-2.09)	12.92/0.56 (0.00-6.28)	0.03/0.00 (0.00-0.00)	0.09/0.00 (0.00-0.00)	0.08/0.00 (0.00-0.00)	0.09/0.00 (0.00 - 0.00)	3.84/0.52 (0.00-2.21)	13.10/0.73 (0.00-6.53)
**3**	6.80/0.73 (0.00-4.97)	12.27/1.37 (0.00-5.91)	0.04/0.00 (0.00-0.00)	0.09/0.00 (0.00-0.00)	0.37/0.00 (0.00-0.00)	0.10/0.00 (0.00-0.00)	7.21/0.79 (0.00-5.23)	12.47/1.55 (0.00-6.11)
**4**	11.39/1.57 (0.00-11.19)	9.58/1.72 (0.00-4.56)	0.07/0.00 (0.00-0.00)	0.09/0.00 (0.00-0.00)	0.72/0.00 (0.00-0.00)	0.09/0.00 (0.00-0.00)	12.19/1.72 (0.00-11.82)	9.75/1.89 (0.00-4.76)
**5 (least deprived)**	10.42/1.57 (0.00-9.86)	9.18/1.62 (0.00-4.25)	0.08/0.00 (0.00-0.00)	0.10/0.00 (0.00-0.00)	0.54/0.00 (0.00-0.00)	0.11/0.00 (0.00-0.00)	11.05/1.74 (0.00-10.35)	9.38/1.78 (0.00-4.47)
**England**	6.83/0.64 (0.00-4.19)	10.61/1.13 (0.00-4.99)	0.05/0.00 (0.00-0.00)	0.09/0.00 (0.00-0.00)	0.35/0.00 (0.00-0.00)	0.09/0.00 (0.00-0.00)	7.22/0.66 (0.00-4.41)	10.79/1.31 (0.00-5.20)
**Kruskal Wallis H-value (degrees of freedom)**	H(4) = 2001 P<0.001	H(4) = 788 P<0.001	H(4) = 560 P<0.001	H(4) = 560 P<0.001	H(4) = 1428 P<0.001	H(4) = 1430 P<0.001	H(4) = 2049 P<0.001	H(4) = 714 P<0.001

For peer review only

Table 2 shows the percentage of heritage sites at risk by EIMD income quintile. Around 1.2% (n=4832) of heritage sites (i.e., 2098 listed buildings, 2639 scheduled monuments and 95 historic parks/gardens) were deemed as 'Heritage at Risk' (HAR). Over 2% in total were found in the most deprived fifth of areas, compared to 1% in Q5 (and between 1.1% and 1.4% in the other quintiles). The most deprived areas had the highest percentage of HAR sites across two of the three categories (2.0% of listed buildings, 21.7% of scheduled monuments). In general, the percentage of HAR listed buildings and scheduled monuments increased as areas became more income deprived. The least deprived areas (Q5) exhibited the highest percentage of historic parks/gardens as 'at risk', i.e., 4.1% of sites compared to 3.4% of sites in Q1.

Table 2. Percentage of heritage sites as 'Heritage at Risk' (2016) (HAR) by EIMD income (2015) quintile.

	**Percentage as HAR**			
**EIMD Income**	**Listed buildings**	**Historic Parks/gardens**	**Scheduled Monuments**	**All sites at risk**
**1 (most deprived)**	2.0	3.4	21.7	2.3
**2**	0.8	2.7	16.4	1.1
**3**	0.6	3.8	15.4	1.4
**4**	0.4	3.0	13.2	1.2
**5 (least deprived)**	0.3	4.1	12.6	1.0
**England**	0.6	3.5	13.8	1.2

Of 30,431 respondents, 59.9% had at least one heritage site within their LSOA, and 61% had visited heritage in the past year (n=19,232). Table 3 shows the findings of the multilevel logistic regression analysis as adjusted odds of visiting heritage by exposure to heritage sites. Respondents with at least one heritage site of any type in their neighbourhood (LSOA) were significantly more likely to have visited a heritage site in the past year (Odds Ratio (OR) 1.12 (95% CI: 1.03-1.22)), than those with no sites (p=0.008). Those with five or more heritage sites per 1000 population, or per km², in their LSOA were most likely to have visited a heritage site (OR 1.16 (95% CI: 1.05-1.30) and 1.14 (95% CI: 1.02-1.26) respectively). The presence of scheduled monuments within respondents' neighbourhoods did not increase the probability of visiting monuments specifically, however with an increase in LSOA-level historic parks/gardens per area the likelihood of visiting such sites increased (OR 1.12 (95% CI: 1.04-1.21), p<0.01) (results not shown).

For peer review only

Table 3. Adjusted odds of visiting heritage by measures of exposure to heritage. (Adjusted by household type, sex, age, income deprivation, ethnicity, education, socio-economic status, housing tenure & health condition/mobility)

		**Odds of visiting heritage**	
		**OR (95%CI)**	**p-value**
**Heritage (any)**	**None**	1.00	*0.008*
	**1+**	*1.12 (1.03-1.22)*	
**Heritage (any) per 1000 pop**	**None**	1.00	*0.014*
	**1-4**	*1.10 (1.00-1.20)*	
	**5+**	*1.16 (1.05-1.30)*	
**Heritage (any) per km²**	**None**	1.00	*0.027*
	**1-4**	*1.11 (1.01-1.22)*	
	**5+**	*1.14 (1.02-1.26)*	

When evaluating associations between mental health and exposure to heritage, interaction effects between heritage exposure and heritage visits were found to be associated with mental health. Table 4 presents the predicted probabilities of poorer mental health by heritage exposure and visits to heritage. Post-hoc testing (with Šidák adjustments for multiple comparisons) found the following associations: Amongst those with any heritage, visitors to heritage had a lower predicted probability of distress (0.171 (95% CI: 0.162-0.179)) compared to non-visitors (0.238 (95% CI: 0.225-0.252)) (Šidák adjusted p-value <0.001). This was also true when considering different levels of exposure, i.e. five or more sites per 1000 population (visitors: 0.165 (95% CI: 0.152-0.177), non-visitors: 0.241 (95% CI: 0.219-0.264), (Šidák p=0.008)), or per km² (visitors: 0.171 (95% CI: 0.158-0.184), non-visitors: 0.246 (95% CI: 0.226-0.267), (Šidák p=0.016)). We found similar results when 'built' heritage (i.e., listed buildings/scheduled monuments only) was included as the exposure variable (controlling for any green space). Amongst those with one or more built heritage sites, or with five or more per 1000 population, visitors had a lower predicted probability of distress. In the former, visitors: 0.171 (95% CI 0.163-0.179), non-visitors: 0.237 (95% CI 0.224-0.251) (Šidák p = 0.001) and, in the latter, visitors: 0.165 (95% CI 0.152-0.178), non-visitors: 0.242 (95% CI 0.219-0.265) (Šidák p = 0.009) (results not shown). As main effects, there were no associations between heritage exposure and distress (GHQ-12), regardless of exposure measure used (p-values: 0.055 – 0.224).

For peer review only

Table 4. Predicted probabilities and 95% confidence intervals (CI) of poorer mental health (GHQ12) for the interaction effects of heritage exposure and visits to heritage. (Adjusted by household type, sex, age, income deprivation, ethnicity, education, socio-economic status, housing tenure, health condition/mobility, exposure to other green space & visits to heritage).

		Non-visitors (95% CI)	Visitors (95% CI)	Interaction p-value
**Heritage**	**None**	0.218 (0.204-0.233)	0.184 (0.174-0.195)	*p=0.005*
	**1+**	*0.238 (0.225-0.252)	*0.171 (0.162-0.179)	
**Heritage per 1000 population**	**None**	0.219 (0.205-0.234)	0.185 (0.174-0.196)	*p=0.011*
	**1-4**	0.235 (0.218-0.251)	0.173 (0.163-0.184)	
	**5+**	**0.241 (0.219-0.264)	**0.165 (0.152-0.177)	
**Heritage per area (1 km²)**	**None**	0.219 (0.205-0.234)	0.185 (0.174-0.195)	*p=0.014*
	**1-4**	0.230 (0.213-0.248)	0.169 (0.159-0.179)	
	**5+**	***0.246 (0.226-0.267)	***0.171 (0.158-0.184)	

Post-hoc multiple comparisons: *Difference p<0.001, **Difference p<0.01, ***Difference p<0.05.

Discussion

This is the first study to explore the spatial distribution of designated heritage sites across areas of varying deprivation in England, and associations between exposure and visits, and mental health. We found that, compared to wealthier areas, the poorest areas had fewer sites per population, and, of these sites, a higher proportion were deemed 'at risk'. Neighbourhood exposure to heritage was associated with visits to heritage; however, the physical presence of neighbourhood heritage, on its own, was not associated with residents' mental health. The combination of having heritage present in the neighbourhood *and* visiting heritage (whether within the neighbourhood or further afield), was associated with better mental health.

Regarding the social-spatial patterning of heritage, the distribution of heritage varied according to how heritage exposure was measured. The most deprived areas showed the lowest mean heritage sites *per population* (Q1:1.83 per 1000), and the least deprived areas the highest (Q4: 12.2 per 1000, Q5: 11.1 per 1000), however, mean sites *per area* for the least (Q1: 9.2 per km²) and most deprived areas (Q5: 9.4 per km²) were similar. This apparent contradiction in findings between measures likely reflects wealthier areas being on average larger in size. In other words, they do have more heritage sites per population, but these are spread across larger geographical extents (LSOA size increase with increasing wealth, see supplemental table 1). Indeed, greater distance to a local heritage site

For peer review only

may be less of a spatial barrier to those on higher incomes, whom are more likely to own cars [35]. Little existing research investigates spatial distribution of heritage on a national scale. A US city-based study, found that higher income/educated neighbourhoods, had superior access to historical and heritage institutions [36]. The authors described spatial access to historical/heritage institutions as 'elitist' with unequal geographic distribution detrimental to poorer neighbourhoods. In our study, perhaps the spatial bias towards higher income areas is a result of heritage increasing overall area wealth. Indeed, conservation area homes in England benefit from price premiums and greater annual price growth [37], and those with the financial means will pay more to live in homes with historic features [38]. We also report that more heritage sites within poorer areas were 'at risk'. HAR sites are more likely to be in a state of disrepair, less aesthetically pleasing, and potentially unsafe, therefore their higher prevalence in poorer neighbourhoods may result in residents being less likely to engage with heritage. Other existing features of poorer neighbourhoods could compound this, such as higher environmental 'incivility' e.g. graffiti, litter, noise and air pollution [39], and vacant and derelict land [40]. It is conceivable that HAR's greater presence within lower income areas, is a result of inequity in the geographical distribution of funding; the Levelling Up paper reports the need for investment in culture and heritage outside London, and within poorer areas [41].

The finding that respondents were more likely to have visited a heritage site in the past year, if they were exposed to local heritage, is comparable to previous evidence on spatial access and attendance at museums or galleries in London [18]. Brook et al. (2016) found that attendance rate variation could not be explained solely by people's individual characteristics and better spatial access was an important contributory factor. Authors concluded that well-resourced locales could provide significant "opportunity structures" for cultural engagement [18]. Certainly better spatial access to other types of environmental features is associated with higher usage, such as sports facilities [42], and green space [43]. We found that those with a higher level of historic parks/gardens locally were more likely to have visited this type of green space in the past year. We cannot say why greater exposure increased the likelihood of heritage visits more generally, or whether local exposure equates to higher local site usage in particular. However, it is feasible that nearby heritage could be a significant 'pull factor' as reduced time and effort may be needed for visits, or visibility of local heritage may contribute to social norms and increase interest in visits, whether near or far.

In terms of a main effect, we found no association between heritage exposure and psychological distress. Associations between heritage engagement and better mental health and wellbeing are established [5-7 9 13 17], however, few studies focus on the mental health benefits of spatial exposure to heritage specifically. The lack of association, in our study, could be attributed to use of proximity as a 'proxy' for engagement with local heritage, while, in reality, location of visits was

For peer review only - http://bmjopen.bmj.com/site/about/guidelines.xhtml

unknown. Future surveys could gather data on how far people travel to engage with heritage. We acknowledge that proximity does not indicate use, or awareness of, local heritage. Residents may lack awareness of local neighbourhood features, such as green space [44] or general amenities [45], and this could be true of local heritage. We did find an interaction effect, amongst those with heritage locally; visitors to heritage showed lower psychological distress. A European-based study looked at associations between neighbourhood green/blue space and mental health [46], and found that although people in highly green/coastal areas experienced better mental health, this relationship did not remain when controlling for recreational visits to these areas. Visit frequency appeared to mediate associations between green/blue space and wellbeing. Authors reasoned that better mental health amongst those in the greenest/bluest areas could be a result of such environmental qualities encouraging visits. Similarly, we found that a combination of proximal heritage, and visits to heritage, was associated with better mental health; higher spatial exposure appeared to provide a pathway to encourage visits. Our findings contribute to discussions about which type of neighbourhood exposure is key in associations with mental health. Many neighbourhood effects' studies define 'exposure' as 'residential proximity', e.g. green space [47]. It is valuable to examine both spatial exposure, and direct contact/engagement, when considering mental health. The beneficial association between greenspace and mental health has been well researched, with theories of benefit based on visualising the structure of greenspace, its colours and its landscapes, to engage the human brain, and work to reduce stress, restore attention, and enhance wellbeing [48]. Less is known about how 'bricks and mortar' heritage influences mental health. We found that, regardless of green space exposure, those with built heritage in their local area, who had visited heritage, showed lower distress. Historic buildings are considered to improve neighbourhood quality with older buildings creating feelings of grandeur and permanence [38], and exposure to urban environments with historic features, has been perceived more restorative, attractive and interesting than those without [49 50]. Viewing heritage appears to engage the senses, and places considered 'special' may stimulate the brain cells that manage emotion [51]. Residents who live in areas with higher proportions of listed buildings, or who regard a building as 'special' within their local area, have a greater 'sense of place' [10] which, in turn, can be associated with better mental health [52]. Regarding local heritage as 'special' could thus be part of the pathway connecting heritage to wellbeing.

Strengths and limitations

Our research exhibited a number of strengths. We used a sample of adults from across the whole of England, weighted to ensure representativeness to the wider population. We explored associations between exposure to heritage, and to visits, and to mental health, within relatively small areas

For peer review only

across the whole of England, while other papers did not incorporate spatial data or nation-wide analyses. We explored whether mental health benefits of ’built’ heritage remained regardless of the presence of green space in the local neighbourhood, providing a unique strand to this research. Regarding limitations, this study was cross-sectional; we cannot assume that key variables demonstrate causal associations, and that findings are generalisable within other contexts. We acknowledge that use of LSOA-level analysis may lead to statistical bias from using arbitrarily classified units to report spatial patterning [53]. We may be limited by the simplified nature of the UKHLS questions on visits to heritage, e.g., those visiting a ‘A city or town with historic character’, may have done so for another purpose, i.e., for every-day activities related to employment, education, food shopping etc. Unfortunately, more detailed heritage visits data that can be linked to spatial data is not available. Doing every-day activities within a heritage environment may be associated with better mental health, however researching this is beyond the scope of our current study. Our study explores spatial exposure to heritage only, and does not incorporate other factors, which may promote or inhibit accessibility, such as financial access, e.g., admittance fees. Although heritage may often be viewed without admittance. We acknowledge the potential for missing HAR data; lack of resources may result in systematic surveys of different HAR types being undertaken at different times [54]. Additionally, we acknowledge that depressed individuals may be less likely to visit heritage due to mental ill health reducing their drive to engage with heritage.

Policy implications

Our findings of inequities in heritage exposure, as a barrier to heritage engagement, are directly relevant to the UK Government’s levelling up agenda. The bill recommends ensuring protection to existing heritage, tackling disparities in heritage access, and improving cultural investment outside London [41]. Going forward the levelling up agenda must address inequalities in heritage exposure as part of a pathway to improve societal mental health. Methods to increase exposure could include the development within areas with fewer heritage sites, of better options for public transport, e.g. subsidised transport to areas with better exposure [36]. Exposure could be improved via investment in heritage in need of reinvigoration, such as at risk sites. Schemes which encourage volunteering to improve at risk assets provide various social, psychological and health benefits to volunteers [55]. Increasing heritage engagement opportunities must consider both geographical and socio-cultural factors [18]. Policy objectives to promote heritage engagement across all cultural groups should seek to redress potentially lower levels of interest among specific groups, such as poorer households [17]. Heritage organisations could expand ‘public outreach’ activities to neighbourhoods and communities with lower exposure [36], or with lower levels of heritage awareness, and compensate for fewer heritage assets in deprived areas, through increased awareness of what *is* there.

For peer review only

Conclusion

We demonstrated that more income-deprived neighbourhoods in England had lower levels of heritage exposure. Exposure to local heritage increased the likelihood of visiting heritage in general, and exposure to any heritage, or built heritage specifically, was positively associated with mental health, but only amongst those who had visited heritage in the past year. Our findings indicate that both spatial exposure and engaging with heritage through visiting are key. There is much to be gained from improving the structure of exposure. Formulation of strategies for schemes to tackle inequality in exposure in poorer neighbourhoods is necessary. Schemes could include working with communities and areas to improve access, knowledge, and awareness of heritage, and providing investment in 'at risk' heritage and currently non-accessible sites. Such schemes could promote local heritage as 'special', to improve heritage engagement, and ultimately provide benefits to mental health and well-being.

Acknowledgements: We wish to thank Neil Guiden (Data & Analysis Manager) at Historic England for supplying spatial data. We wish to acknowledge the UKHLS participants for their involvement in the study and the UK Data Service for supplying all social survey data used.

Authors contributors: LMac, EG, LM, and RM proposed the research questions. LMac led the project, created GIS variables, and drafted all versions of the article. NN undertook statistical analysis and interpretation. All authors critically revised the article, read and approved final draft.

Funding: LMac, NN and RM are funded by the UK Medical Research Council (MRC) Places and Health Programme (MC_UU_00022/4) and the Chief Scientist Office (CSO) (SPHSU2019) at the MRC/CSO Social and Public Health Sciences Unit, University of Glasgow, UK.

Competing interests: The authors declare no conflict of interest.

Patient consent for publication: Not required.

Ethics approval: The University of Essex Ethics Committee has approved all data collection on Understanding Society. The UKHLS main study gained consent for all data linkages (except to health records) and respondents aged 16 or over provided informed and written consent to participate.

Provenance and peer review: Not commissioned; externally peer reviewed.

Data availability statement: Data may be obtained from a third party and are not publicly available.

Supplemental material: This content has been supplied by the authors.

For peer review only

Open access: This is an open access article distributed in accordance with the Creative Commons Attribution 4.0 Unported (CC BY 4.0) license, which permits others to copy, redistribute, remix, transform and build upon this work for any purpose, provided the original work is properly cited, a link to the licence is given, and indication of whether changes were made. See: https://creativecommons.org/licenses/by/ 4.0/.

ORCID: Laura Macdonald https://orcid.org/0000-0002-0593-8079

References

1. Jimenez MP, DeVille NV, Elliott EG, *et al.* Associations between nature exposure and health: A review of the evidence. *International Journal of Environmental Research and Public Health* 2021;18:4790.
2. Twohig-Bennett C, Jones A. The health benefits of the great outdoors: A systematic review and meta-analysis of greenspace exposure and health outcomes. *Environmental research* 2018;166:628-37.
3. Ward Thompson C, Silveirinha de Oliveira E, Wheeler BW, *et al.* Urban Green Spaces and Health, A Review of Evidence. Copenhagen: World Health Organisation, 2016.
4. Historic England. About The List. 2022. https://historicengland.org.uk/listing/the-list/about-the-list/ [Accessed 27 June 2022].
5. Brown JL, MacDonald R, Mitchell R. Are People Who Participate in Cultural Activities More Satisfied with Life? *Social Indicators Research* 2015;122:135-46.
6. Fujiwara D, Cornwall T, Dolan P. Heritage and Wellbeing. London: English Heritage, 2014.
7. Leadbetter C, O'Connor N. Healthy Attendance? The Impact of Cultural Engagement and Sports Participation on Health and Satisfaction with Life in Scotland. Edinburgh: Scottish Government, 2013.
8. Lakey J, Smith N, Oskala A, *et al.* Culture, sport and well-being. Findings from the Understanding Society Youth Survey. London: NatCen Social Research, 2017.
9. Mak HW, Coulter R, Fancourt D. Associations between community cultural engagement and life satisfaction, mental distress and mental health functioning using data from the UK Household Longitudinal Study (UKHLS): are associations moderated by area deprivation? *BMJ open* 2021;11:e045512.
10. Bradley DP, Coombes M, Tranos E, *et al.* Sense of place and social capital and the historic built environment: Report of research for English Heritage. Newcastle: Newcastle University and Bradley Research and Consulting, 2009.
11. Harris PB, Werner CM, Brown BB, *et al.* Relocation and privacy regulation: A cross-cultural analysis. *Journal of environmental psychology* 1995;15:311-20.
12. Rollero C, De Piccoli N. Does place attachment affect social well-being? *European Review of Applied Psychology* 2010;60:233-38.
13. Darvill T, Heaslip V, Barrass K. Heritage and well-being: Therapeutic places past and present. In: Darvill T, Barrass K, Drysdale L, et al., eds. Historic Landscapes and Mental Well-being. Oxford: Archaeopress, 2019:17.
14. Grossi E, Tavano Blessi G, Sacco PL. Magic moments: determinants of stress relief and subjective wellbeing from visiting a cultural heritage site. *Culture, Medicine, and Psychiatry* 2019;43:4-24.
15. Sofaer J, Davenport B, Sørensen MLS, *et al.* Heritage sites, value and wellbeing: learning from the COVID-19 pandemic in England. *International Journal of Heritage Studies* 2021;27:1117-32.

For peer review only

16. National Statistics. Taking Part 2019/20 - Heritage. 2020. https://www.gov.uk/government/statistics/taking-part-201920-heritage [Accessed 08 February 2022].
17. Lakey J, Smith N, Oskala A*, et al.* Culture, sport and well-being. Findings from the Understanding Society Adult Survey. London: NatCen Social Research, 2017.
18. Brook O. Spatial equity and cultural participation: how access influences attendance at museums and galleries in London. *Cultural Trends* 2016;25:21-34.
19. OCSI. 'Left behind' Neighbourhoods: Community data dive. 2021. https://www.appg-leftbehindneighbourhoods.org.uk/wp-content/uploads/2021/06/APPG-Community-Data-Dive-Report-for-APPG-S7.pdf [Accessed 09 February 2022].
20. HM Government. Levelling Up the United Kingdon. London: Her Majesty's Stationery Office, 2022.
21. Office for National Statistics. Census geography. 2021. https://www.ons.gov.uk/methodology/geography/ukgeographies/censusgeography [Accessed 09 May 2022].
22. UK Government. English indices of deprivation 2015. 2015. https://www.gov.uk/government/statistics/english-indices-of-deprivation-2015 [Accessed 20 October 2021].
23. Office for National Statistics. Lower layer Super Output Area population estimates. 2021. https://www.ons.gov.uk/peoplepopulationandcommunity/populationandmigration/populationestimates/datasets/lowersuperoutputareamidyearpopulationestimates [Accessed 20 October 2021].
24. Historic England. What is the Heritage at Risk Programme? 2022. https://historicengland.org.uk/advice/heritage-at-risk/types/ [Accessed 21 March 2022].
25. Ordnance Survey. OS Open Greenspace. 2021. https://www.ordnancesurvey.co.uk/business-government/products/open-map-greenspace.
26. Office for National Statistics. Lower Layer Super Output Area (LSOA) boundaries. 2016. https://data.gov.uk/dataset/fa883558-22fb-4a1a-8529-cffdee47d500/lower-layer-super-output-area-lsoa-boundaries.
27. Kaminska O, ., Lynn P. Weighting and Sample Representation. London: Institute for Social and Economic Research, University of Essex, 2019.
28. Goldberg DP. User's guide to the General Health Questionnaire. Windsor, 1988.
29. Goldberg DP, Oldehinkel T, Ormel J. Why GHQ threshold varies from one place to another. *Psychological medicine* 1998;28:915-21.
30. Astell-Burt T, Mitchell R, Hartig T. The association between green space and mental health varies across the lifecourse. A longitudinal study. *Journal of Epidemiology and Community Health* 2014;68:578-83.
31. Mak HW, Coulter R, Fancourt D. Associations between neighbourhood deprivation and engagement in arts, culture and heritage: evidence from two nationally-representative samples. *BMC public health* 2021;21:1-10.
32. Von Elm E, Altman DG, Egger M*, et al.* The Strengthening the Reporting of Observational Studies in Epidemiology (STROBE) statement: guidelines for reporting observational studies. *Annals of internal medicine* 2007;147:573-77.
33. UKHLS. Clustering and stratification. 2022. https://www.understandingsociety.ac.uk/documentation/mainstage/user-guides/main-survey-user-guide/clustering-and-stratification [Accessed 11 January 2023].
34. StataCorp. Stata Statistical Software. College Station, Texas: StataCorp LLC, 2021.
35. Department for Transport. Travel by vehicle availability, income, ethnic group, household type, mobility status and NS-SEC. 2020. https://www.gov.uk/government/statistical-data-sets/nts07-car-ownership-and-access#full-publication-update-history [Accessed 31 May 2022].

For peer review only

36. Moldavanova AV, Meloche L, Thompson TL. Understanding the geography of access to cultural amenities: The case of Metropolitan Detroit. *Journal of Urban Affairs* 2021:1-26.
37. Ahlfeldt GM, Holman N, Wendland N. An assessment of the effects of conservation areas on value. London: London School of Economics and Political Science, 2012.
38. Maeer G, Robinson A, Hobson M. Values and benefits of heritage A research review. London: Heritage Lottery Fund, 2016.
39. Braubach M, Fairburn J. Social inequities in environmental risks associated with housing and residential location—a review of evidence. *European journal of public health* 2010;20:36-42.
40. Macintyre S, Macdonald L, Ellaway A. Do poorer people have poorer access to local resources and facilities? The distribution of local resources by area deprivation in Glasgow, Scotland. *Social science & medicine* 2008;67:900-14.
41. The Heritage Alliance. Levelling Up White Paper Summary for Heritage. London: The Heritage Alliance, 2022.
42. Giles-Corti B, Donovan RJ. The relative influence of individual, social and physical environment determinants of physical activity. *Social Science & Medicine* 2002;54:1793–812.
43. Pyky R, Neuvonen M, Kangas K*, et al.* Individual and environmental factors associated with green exercise in urban and suburban areas. *Health & place* 2019;55:20-28.
44. Macintyre S, Macdonald L, Ellaway A. Lack of agreement between measured and self-reported distance from public green parks in Glasgow, Scotland. *International Journal of Behavioral Nutrition and Physical Activity* 2008;5:1-8.
45. Macdonald L, Kearns A, Ellaway A. Do residents' perceptions of being well-placed and objective presence of local amenities match? A case study in West Central Scotland, UK. *BMC Public Health* 2013;13:1-11.
46. White MP, Elliott LR, Grellier J*, et al.* Associations between green/blue spaces and mental health across 18 countries. *Scientific reports* 2021;11:1-12.
47. Bratman GN, Hamilton JP, Daily GC. The impacts of nature experience on human cognitive function and mental health. *Annals of the New York Academy of Sciences* 2012;1249:118–36.
48. Markevych I, Schoierer J, Hartig T*, et al.* Exploring pathways linking greenspace to health: Theoretical and methodological guidance. *Environmental research* 2017;158:301-17.
49. Bornioli A, Parkhurst G, Morgan PL. Psychological wellbeing benefits of simulated exposure to five urban settings: an experimental study from the pedestrian's perspective. *Journal of transport & health* 2018;9:105-16.
50. Bornioli A, Parkhurst G, Morgan PL. The psychological wellbeing benefits of place engagement during walking in urban environments: A qualitative photo-elicitation study. *Health & place* 2018;53:228-36.
51. National Trust. Why Places Matter to People Research Report. Wiltshire: National Trust, 2018.
52. Williams A, Kitchen P. Sense of Place and Health in Hamilton, Ontario: A Case Study. *Social indicators research* 2012;108:257-76.
53. Openshaw S. The Modifiable Areal Unit Problem. Norwich, England: Geobooks, 1984.
54. Historic England. Heritage at Risk Statistics: Method Statement. London: Historic England, 2021.
55. Lewis C, Siriwardena N, Laparidou D*, et al.* Wellbeing in Volunteers on Heritage at Risk Projects. London: Historic England, 2021.

For peer review only

Figure 1. Flow chart summary of GIS and individual data used

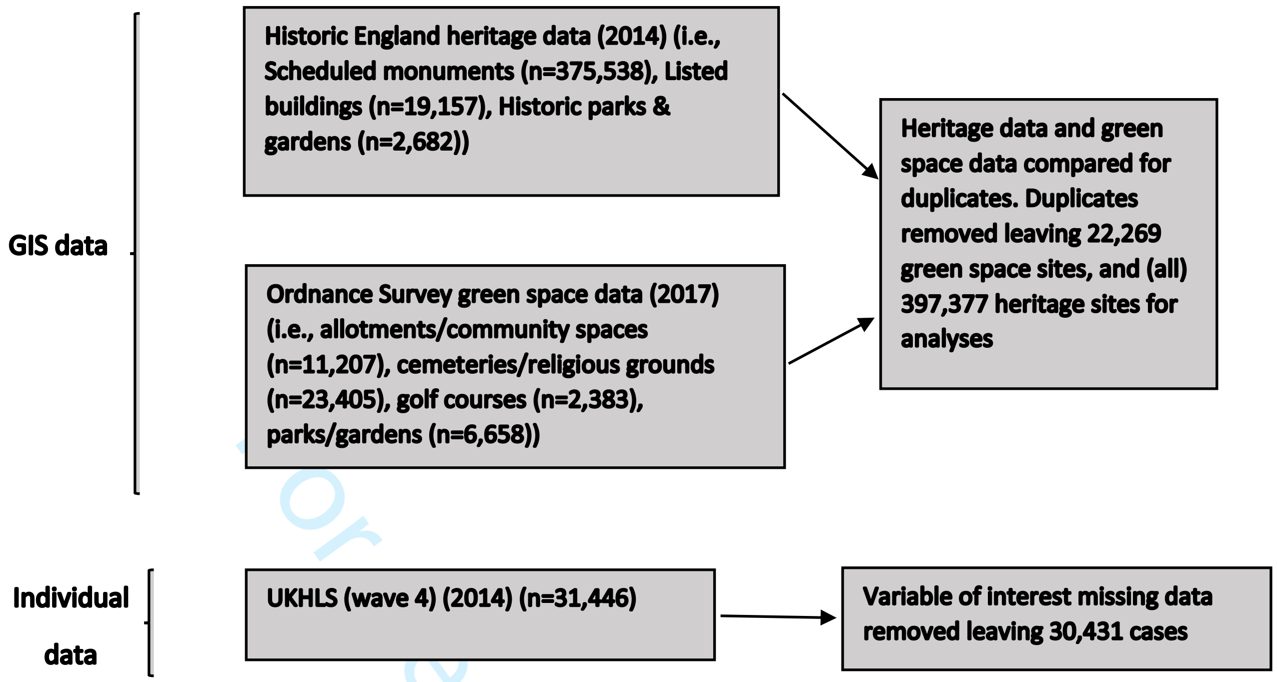

Supplemental table 1. EIMD Income Quintile descriptive – population, geographical area, heritage.

EIMD Income	Population (Mean, SD)	Area km² (Mean, SD)	Listed buildings (N)	Historic Parks/ Gardens (N)	Scheduled Monuments (N)	All sites (N)
**1 (most deprived)**	11,075,909 (1,686, 331.36)	3,428 (0.52, 1.19)	19,880	203	277	20,360
**2**	11,149,857 (1,697, 362.87)	7,547 (1.15, 3.71)	41,189	292	913	42,394
**3**	11,016,312 (1,677, 360.18)	26,114 (3.98, 12.42)	75,084	470	4,170	79,724
**4**	10,820,663 (1,647, 355.98)	50,252 (7.65, 20.69)	125,462	790	7,904	13,4156
**5 (least deprived)**	10,723,586 (1,632, 407.47)	43,089 (6.56, 15.89)	113,923	927	5,893	120,743
**Total**	54,786,327 (1,668, 365.19)	130,432 (3.97, 13.34)	375,538	2,682	19,157	397,377

Supplemental table 2. Complete case sample descriptive (percentage by each category)

(base n=30420)

**Age**	<20 years old	6.1
	20-29 years old	12.6
	30-39 years old	15.9
	40-49 years old	18.9
	50-59 years old	16.9
	60-69 years old	15.3
	70+ years old	14.4
	Missing	0
**Sex**	Male	44.6
	Females	55.4
	Missing	0

For peer review only

**EIMD income**	Q1 (most deprived)	20.5
	Q2	19.3
	Q3	20.6
	Q4	20.7
	Q5 (least deprived)	18.9
	Missing	0
**Ethnicity**	White	82.5
	Other	17.5
	Missing	21
**Education**	Degree or higher	36.4
	GCSEs/A-levels	41.8
	Other qualification	9.6
	No qualification	12.3
	Missing	54
**Job type (NS-SEC)**	Managerial, admin & professional	24.7
	Intermediate occupations	13.7
	Routine & manual occupations	19.8
	Not in paid employment	41.9
	Missing	341
**Housing tenure**	Owner occupier	71.3
	Social renter	16.7
	Private renter	12.1
	Missing	499
**Long-term illness/mobility**	No long-term illness or mobility issue	53.0
	Long-term illness	14.8
	Long-term illness and mobility issue	32.2
	Missing	11

For peer review only

**Marital/cohabitating status**	Single	21.8
	Married/cohabiting	63.7
	Divorced/separated	8.6
	Widowed	5.9
	Missing	70

For peer review only

Reporting checklist for cross sectional study.

Based on the STROBE cross sectional guidelines.

Instructions to authors

Complete this checklist by entering the page numbers from your manuscript where readers will find each of the items listed below.

Your article may not currently address all the items on the checklist. Please modify your text to include the missing information. If you are certain that an item does not apply, please write "n/a" and provide a short explanation.

Upload your completed checklist as an extra file when you submit to a journal.

In your methods section, say that you used the STROBE cross sectionalreporting guidelines, and cite them as:

von Elm E, Altman DG, Egger M, Pocock SJ, Gotzsche PC, Vandenbroucke JP. The Strengthening the Reporting of Observational Studies in Epidemiology (STROBE) Statement: guidelines for reporting observational studies.

		Reporting Item	Page Number
**Title and abstract**			
Title	#1a	Indicate the study's design with a commonly used term in the title or the abstract	2
Abstract	#1b	Provide in the abstract an informative and balanced summary of what was done and what was found	2

For peer review only

Introduction

Background / rationale	#2	Explain the scientific background and rationale for the investigation being reported	3-5
Objectives	#3	State specific objectives, including any prespecified hypotheses	5

Methods

Study design	#4	Present key elements of study design early in the paper	5-9
Setting	#5	Describe the setting, locations, and relevant dates, including periods of recruitment, exposure, follow-up, and data collection	5-9
Eligibility criteria	#6a	Give the eligibility criteria, and the sources and methods of selection of participants.	8
	#7	Clearly define all outcomes, exposures, predictors, potential confounders, and effect modifiers. Give diagnostic criteria, if applicable	5-9
Data sources / measurement	#8	For each variable of interest give sources of data and details of methods of assessment (measurement). Describe comparability of assessment methods if there is more than one group. Give information separately for for exposed and unexposed groups if applicable.	5-9
Bias	#9	Describe any efforts to address potential sources of bias	5-9

For peer review only

Study size	#10	Explain how the study size was arrived at	5-9
Quantitative variables	#11	Explain how quantitative variables were handled in the analyses. If applicable, describe which groupings were chosen, and why	5-9
Statistical methods	#12a	Describe all statistical methods, including those used to control for confounding	8-9
Statistical methods	#12b	Describe any methods used to examine subgroups and interactions	8-9
Statistical methods	#12c	Explain how missing data were addressed	9
Statistical methods	#12d	If applicable, describe analytical methods taking account of sampling strategy	n/a
Statistical methods	#12e	Describe any sensitivity analyses	n/a
**Results**			
Participants	#13a	Report numbers of individuals at each stage of study—eg numbers potentially eligible, examined for eligibility, confirmed eligible, included in the study, completing follow-up, and analysed. Give information separately for for exposed and unexposed groups if applicable.	n/a
Participants	#13b	Give reasons for non-participation at each stage	n/a
Participants	#13c	Consider use of a flow diagram	n/a

For peer review only

Descriptive data	#14a	Give characteristics of study participants (eg demographic, clinical, social) and information on exposures and potential confounders. Give information separately for exposed and unexposed groups if applicable.	(supplemental table)
Descriptive data	#14b	Indicate number of participants with missing data for each variable of interest	(supplemental table)
Outcome data	#15	Report numbers of outcome events or summary measures. Give information separately for exposed and unexposed groups if applicable.	n/a
Main results	#16a	Give unadjusted estimates and, if applicable, confounder-adjusted estimates and their precision (eg, 95% confidence interval). Make clear which confounders were adjusted for and why they were included	9-12
Main results	#16b	Report category boundaries when continuous variables were categorized	7
Main results	#16c	If relevant, consider translating estimates of relative risk into absolute risk for a meaningful time period	n/a
Other analyses	#17	Report other analyses done—e.g., analyses of subgroups and interactions, and sensitivity analyses	9-10

Discussion

For peer review only

Key results	#18	Summarise key results with reference to study objectives	13
Limitations	#19	Discuss limitations of the study, taking into account sources of potential bias or imprecision. Discuss both direction and magnitude of any potential bias.	15
Interpretation	#20	Give a cautious overall interpretation considering objectives, limitations, multiplicity of analyses, results from similar studies, and other relevant evidence.	13-16
Generalisability	#21	Discuss the generalisability (external validity) of the study results	15
**Other Information**			
Funding	#22	Give the source of funding and the role of the funders for the present study and, if applicable, for the original study on which the present article is based	17

Notes:

- 14a: 15 (supplementary table)
- 14b: 15 (supplementary table)
- 16a: 9-10 (and tables) The STROBE checklist is distributed under the terms of the Creative Commons Attribution License CC-BY. This checklist was completed on 26. July 2022 using https://www.goodreports.org/, a tool made by the EQUATOR Network in collaboration with Penelope.ai

For peer review only

Is spatial exposure to heritage associated with visits to heritage and to mental health? A cross-sectional study using data from the UK Household Longitudinal Study (UKHLS).

Journal:	*BMJ Open*
Manuscript ID	bmjopen-2022-066986.R2
Article Type:	Original research
Date Submitted by the Author:	01-Mar-2023
Complete List of Authors:	Macdonald, Laura; University of Glasgow MRC/CSO Social and Public Health Sciences Unit, MRC Social and Public Health Sciences Unit; University of Glasgow MRC/CSO Social and Public Health Sciences Unit,<br>Nicholls, Natalie; University of Glasgow MRC/CSO Social and Public Health Sciences Unit<br>Gallou, Eirini; Historic England, Policy and Evidence<br>Monckton, Linda; Historic England, Policy and Evidence<br>Mitchell, Richard; University of Glasgow MRC/CSO Social and Public Health Sciences Unit
**Primary Subject Heading**:	Public health
Secondary Subject Heading:	Mental health
Keywords:	MENTAL HEALTH, STATISTICS & RESEARCH METHODS, PUBLIC HEALTH

SCHOLARONE™
Manuscripts

I, the Submitting Author has the right to grant and does grant on behalf of all authors of the Work (as defined in the below author licence), an exclusive licence and/or a non-exclusive licence for contributions from authors who are: i) UK Crown employees; ii) where BMJ has agreed a CC-BY licence shall apply, and/or iii) in accordance with the terms applicable for US Federal Government officers or employees acting as part of their official duties; on a worldwide, perpetual, irrevocable, royalty-free basis to BMJ Publishing Group Ltd ("BMJ") its licensees and where the relevant Journal is co-owned by BMJ to the co-owners of the Journal, to publish the Work in this journal and any other BMJ products and to exploit all rights, as set out in our licence.

The Submitting Author accepts and understands that any supply made under these terms is made by BMJ to the Submitting Author unless you are acting as an employee on behalf of your employer or a postgraduate student of an affiliated institution which is paying any applicable article publishing charge ("APC") for Open Access articles. Where the Submitting Author wishes to make the Work available on an Open Access basis (and intends to pay the relevant APC), the terms of reuse of such Open Access shall be governed by a Creative Commons licence – details of these licences and which Creative Commons licence will apply to this Work are set out in our licence referred to above.

Other than as permitted in any relevant BMJ Author's Self Archiving Policies, I confirm this Work has not been accepted for publication elsewhere, is not being considered for publication elsewhere and does not duplicate material already published. I confirm all authors consent to publication of this Work and authorise the granting of this licence.

For peer review only

Is spatial exposure to heritage associated with visits to heritage and to mental health? A cross-sectional study using data from the UK Household Longitudinal Study (UKHLS).

Laura Macdonald [a], Natalie Nicholls [a], Eirini Gallou [b], Linda Monckton [b], Richard Mitchell [a].

a. MRC/CSO Social and Public Health Sciences Unit, University of Glasgow, Glasgow, UK

b. Policy and Evidence, Historic England, London, UK

Correspondence to:

Mrs Laura Macdonald,

Places and Health Programme,

MRC/CSO Social and Public Health Sciences Unit,

University of Glasgow.

laura.macdonald@glasgow.ac.uk

For peer review only

Abstract

Objectives: Existing research highlights the beneficial nature of heritage engagement for mental health, but engagement varies geographically and socially, and few studies explore spatial exposure (i.e., geographic availability) to heritage and heritage visits. Our research questions were 'does spatial exposure to heritage vary by area income deprivation?', 'is spatial exposure to heritage linked to visiting heritage?', and 'is spatial exposure to heritage linked to mental health?'. Additionally, we explored whether local heritage is associated with mental health regardless of the presence of green space.

Design: Data collected January 2014-June 2015 via the UK Household Longitudinal Study (UKHLS) wave 5. Our study is cross-sectional.

Setting: UKHLS data was either collected via face-to-face interview or on-line questionnaire.

Participants: 30,431 adults (16 years+) (13,676 males, 16,755 females). Participants geocoded to Lower Super Output Area (LSOA) 'neighbourhood' and 'English Index Multiple Deprivation' (EIMD) 2015 Income score.

Main exposures/outcome measures: LSOA-level heritage exposure and green space exposure (i.e., population and area densities); heritage site visit in past year (outcome, binary: no, yes); mental distress (outcome, General Health Questionnaire-12, binary: less distressed 0-3, more distressed 4+).

Results: Heritage varied by deprivation, the most deprived areas (Income Quintile (Q)1:1.8) had fewer sites per 1000 population than the least deprived (Q5:11.1) ($p<0.01$). Compared to those with no LSOA-level heritage, those with heritage exposure were more likely to have visited a heritage site in the past year (OR: 1.12 (95%CI: 1.03-1.22)) ($p<0.01$). Amongst those with heritage exposure, visitors to heritage had a lower predicted probability of distress (0.171 (95%CI: 0.162-0.179)) than non-visitors (0.238 (95%CI: 0.225-0.252)) ($p<0.001$).

Conclusions: Our research contributes to evidence for the wellbeing benefits of heritage and is highly relevant to the government's levelling-up heritage strategy. Our findings can feed into schemes to tackle inequality in heritage exposure to improve both heritage engagement and mental health.

Keywords: heritage, GIS, inequalities, mental health.

For peer review only

Strengths and limitations of this study

- Our paper is the first to explore associations between spatial exposure to heritage, and visits to heritage, and mental health, within relatively small areas across England.
- We use a sample of adults from across England, with sampling weighted to ensure representativeness to the wider population of England.
- We use a variety of LSOA-level measures to capture exposure to heritage, i.e. population and area densities, and percentage of sites as 'at risk'.
- We explore whether associations between mental health and heritage remained regardless of the presence of local green space, providing a unique strand to this research.
- Our study was cross-sectional; thus, we cannot assume that key variables demonstrate causal associations.

Introduction

Population health research has paid much attention to the health benefits of exposure to natural environments [1]. Regular contact with green space has been associated with better physical health, e.g. through reduced risk of hypertension, stroke, cardiovascular disease and asthma, and improved immune function [2]. There is stronger evidence for mental health benefits of time spent in green and natural space, such as improved cognitive function and brain activity [1], greater social cohesion, and sense of community, relaxation and restorative psychological effects, and lower levels of depression, anxiety and stress [3].

A much smaller evidence base suggests that visiting heritage may also benefit mental health and wellbeing. In this study, we define heritage as a building, monument, site or area classified as having 'a degree of significance' worthy of consideration in planning related decisions, due to its architectural or historic interest [4]. Heritage assets may be 'designated' by the Secretary of State for Digital, Culture, Media and Sport (DCMS) as advised by Historic England, or recognised for their significance by local planning authorities, while 'World heritage sites' are further recognised for their international value by bodies such as UNESCO. People who frequently visit heritage sites report higher life satisfaction [5-7], enhanced levels of self-esteem and happiness [8], and lower levels of mental distress [9]. Residents of places with greater numbers of heritage sites have a stronger 'sense of place' [10] and higher levels of place attachment have been associated with better quality of life [11], and 'social wellbeing' [12]). Studies have also shown that engaging with heritage can be psychologically restorative [13], and that visits to cultural heritage sites can reduce stress [14]. The

For peer review only

denial of access to heritage during the Covid-19 lockdowns also provided evidence for its benefits. Awareness of the value of heritage to mental wellbeing was reportedly reinforced by being unable to access it [15].

One problem with studies of both green space and heritage is that they do not often acknowledge the overlap between them, i.e., the potential for co-occurrence of green space and heritage in the same area. Historic parks and gardens are 'cultural assets' in that they are commonly designed or created by and for people with a purpose. They are often cultural landscapes in their own right, but many parks and gardens contain historic buildings or have historic value, and many monuments and buildings are close to or surrounded by green spaces. Furthermore, the parts of towns and cities that contain more (or less) heritage might well also contain more (or less) green space and trying to 'untangle' the contributions of each environment to health and wellbeing is a difficult task. Overall, though, evidence suggests that exposure to heritage has the potential to benefit health, and in particular, mental health.

Despite the potential benefits, visits to heritage are unequal. People living in more deprived areas are less likely to engage in the arts, culture and heritage regardless of personal socio-economic status [9]. In the UK in 2019/2020, a higher proportion of adults resident in the least deprived areas (83%) had visited sites in the past year than those resident in the most deprived (50%) [16]. Heritage engagement levels vary by individual characteristics too with, e.g., those in employment, and in higher socio-economic groups, more likely to visit [17]. Geographical availability of cultural assets, such as museums and libraries is associated with visits or engagement [18], but availability is unequal across the UK with less deprived areas showing better access [19]. In this study, we refer to this geographical availability factor as 'exposure.' There is surprisingly little evidence about exposure to heritage, about how it interacts with the socio-economic characteristics of the population and whether it is implicated in any health benefits. Such evidence is important to both agencies concerned with improving and equalising population health, and those seeking to understand the value and potential of heritage. If levels of exposure make a difference for both engagement and health, there is potential for place-based schemes to improve and equalise it.

To address this gap, in this paper we explore whether spatial exposure to designated heritage (i.e. listed buildings, scheduled monuments and historic parks/gardens) varies by area income deprivation within England, and whether spatial exposure to heritage is linked to visits to heritage (unadjusted and adjusted for socio-demographic factors). We then explore whether exposure to heritage is associated with mental health among all residents, and those who are visitors and non-visitors (controlling for socio-demographic factors and other green space). Additionally, we explore

For peer review only

'built' heritage, specifically, to understand whether exposure to listed buildings and scheduled monuments is associated with mental health benefits beyond those associated with exposure to green space. We consider whether exposure to heritage in an area is related to psychological wellbeing among those living in proximity to it and disentangle the potential overlap in associations with well-being related to heritage exposure and to green space exposure. Our research is timely, with, in the 2022 Levelling Up paper, the UK government showing a commitment to tackling socio-spatial inequalities in heritage access and engagement [20].

In summary, our three research questions are: 'does spatial exposure to heritage (and heritage 'at risk') vary by area deprivation?', 'Is spatial exposure to heritage linked to visiting heritage?', and 'Is spatial exposure to heritage (i.e., any, or 'built' heritage specifically) linked to mental health among heritage visitors and non-visitors?'.

Methods

To answer our research questions, we needed four data components describing: (i) local area population, context and socio-economic situation; (ii) spatial exposure to heritage; (iii) spatial exposure to other green spaces; and (iv) individual level visits to heritage, health, and socio-demographic covariates. These data needed a common spatial framework to represent, and georeference individuals (i.e., residential location related to a ground system of geographic coordinates) to, the 'local area' or 'neighbourhood'. The spatial framework was Lower Super Output Area (LSOA) (as individual addresses or postal codes not available). LSOAs are small area units created for collecting and reporting statistics about the UK population [21]. They are socially homogeneous and consistent in population size; however, their geographical size varies according to level of urban/rural (e.g., rural LSOAs are geographically larger as their populations are more dispersed). LSOAs have a median area size of 0.46 km² and median population size of 1,500 residents or 650 households (see supplemental table 1 for more information). There are 32,844 LSOAs in England and they are commonly used as a proxy for 'neighbourhood'.

Data

(i) local area population, context and socio-economic situation

We obtained the English Index of Multiple Deprivation (EIMD) 2015 (income domain) ranks and quintiles [22] and 2015 mid-year population estimates [23] to describe LSOA population and socio-economic situation. We did not use the full EIMD as it includes barriers to local services and health variables, therefore, there may have been some circularity in investigating whether the full EIMD was associated with access to heritage/green space and/or mental health. An EIMD Income quintile

For peer review only

descriptive is included within supplemental table 1 (including total and mean population/size per quintile).

(ii) and (iii) Spatial exposure to heritage and other green spaces.

Heritage data: We obtained spatial data on all nationally protected historic buildings and sites from Historic England for 2014 [4]. The National Heritage List for England originated in 1882 when the first powers of protection were established and became a statutory ‘Listing’ after World War Two (mid-1940s). Currently, the List holds over 400,000 entries and draws together all scheduled monuments, listed buildings, registered landscapes, battlefields, and protected wrecks. Historic England continuously updates the list, curated from the DCMS [4]. In our analysis we included listed buildings (points only – in compliance with Ordnance Survey (OS) licence), scheduled monuments (polygons), and historic parks/gardens (polygons). We excluded wreck-sites (not visible from land) and excluded battlefields as small numbers of these sites (n=47, sited within 0.1% of LSOAs only) would not allow meaningful comparison across income quintiles. Listed buildings are buildings (e.g., residences, farmhouses, churches etc.) recognised as being of special architectural or historic interest. Scheduled Monuments are historic sites of national importance that are legally protected, such as, Roman remains, burial mounds, castles, bridges, earthworks, etc. Historic parks/gardens are protected 'designed' landscapes, including a range of planned open spaces, such as public parks, cemeteries, private house grounds etc. The dataset also distinguished ‘Heritage at Risk’ (HAR) sites. HAR is an official description of a heritage asset added to the HAR register that is maintained by Historic England, and records the condition of designated heritage assets after assessment. Such sites are vulnerable to loss due to “neglect, decay or inappropriate development” and are in need of safeguarding and protection [24].

Other green space data: We obtained data on other green space for England from the OS Open Greenspace database. Data for 2014 were not available, with the closest temporal match being July 2017 [25]. These were vector data (scale 1:25000) including polygon boundaries of green spaces. We selected green spaces that we considered ‘natural heritage’, including: allotments and community growing spaces; cemeteries and other religious grounds; golf courses; and parks/gardens. We excluded spaces associated with sport/play: tennis courts; bowling greens; other sports facilities; playing fields; and play spaces. Golf courses may be regarded as sports facilities; however, these were included as they are often geographically large and highly visual green spaces, which may contain publicly accessible footpaths and can be surrounded by other green space or coastal walks (i.e., proximal access is available). Some heritage sites included in the data from Historic England were likely to also feature in the OS data. To ensure we did not double count these, we overlaid the

For peer review only

two datasets in a Geographic Information System (GIS), ArcMap 10.8.1, to check for and remove duplicates; any feature captured in the data from Historic England was removed from the OS green space data. The remaining features are referred to as 'other green space'. We obtained LSOA boundaries (2011) [26] and used the 'Overlay' 'Spatial Join' tool to link the points (or polygon centroids/centre points) in the heritage and other green spaces datasets to LSOA boundaries. If a site (polygon) was spread across more than one LSOA, it was linked to the LSOA in which its centroid was located. We then calculated a count of each heritage feature and a count of each other green space feature in each LSOA.

Exposure variables: We then used IBM SPSS statistics v28 to calculate three categorical exposure measures of heritage (at LSOA level). These were 'presence or absence of heritage sites', 'heritage sites per 1000 population', and 'heritage sites per kilometre squared (km^2)'. Using these three measures allowed us to model whether certain 'levels of' heritage were important, and allowed us to account for variation in the geographical size of LSOAs. The per population and per area exposure variables were grouped into 'none', 'low-medium levels (1-4 sites)', and 'higher levels' (five or more sites)'. Due to no existing methodology on appropriate groupings of exposure we based our thresholds on the distribution of values and on percentile representations i.e., numbers roughly represented none, 25th-50th percentile, and 75th percentile, respectively. We also used the same method detailed above to create the per population and per area exposure variables for: 'built' heritage (i.e., listed buildings/scheduled monuments), historic parks/gardens, other green space (non-heritage), and any green space (heritage and non-heritage combined). We joined the area measures of heritage, 'built' heritage, other green spaces, and any green spaces to individual level data via the individual LSOA of residence. UKHLS individuals' LSOA codes were accessed via a UK Data Service special licence.

(iv) individual-level visits to heritage, health, and socio-demographic covariates

Visits to heritage: we undertook a secondary analysis with individual-level data drawn from wave five (2014) of the UK Household Longitudinal Study (UKHLS, 2021) (see supplemental table 2 for sample descriptive). Data were obtained via the UK Data Service. The UKHLS is a large panel survey, which used a clustered and stratified, probability sampling method, and tracked around 31,000 people in England since 2009 (for full details on the survey and its methodology including sampling methods see [27]).We used wave five as it was the most recent wave with questions about visits to heritage. Respondents were asked how often (i.e., 'not once in the last 12 months', 'once in the last 12 months', 'twice in the last 12 months', 'at least 3 or 4 times a year', 'at least once a month', 'at least once a week') they visited each of these heritage sites:-'a city or town with historic character',

For peer review only

‘a historic building’, ‘a historic place of worship’, ‘a historic park or garden, ‘a place of industrial history or historic transport system’, ‘a monument such as a castle, fort or ruin’, ‘a site of archaeological interest’ or a ‘sports heritage site’, in the past year. We did not know whether visits to heritage were in respondent’s LSOA or not.

Mental health measure and covariates: UKHLS carries a General Health Questionnaire (GHQ-12) subjective wellbeing measure, which we included as a measure of mental health. The GHQ-12 includes 12 questions evaluating general mental health functioning and distress. Responses use a 4-point scoring system, i.e. better than normal, same as usual, worse than usual, or much worse than usual, and responses are summed to a score varying from 0 to 12 [28]. We modelled the score as a binary variable lower distress (0-3) and higher distress (i.e., minor psychiatric morbidity)’ (4+); the range of GHQ-12 values was not appropriate for use as a continuous numeric outcome as it is a bounded interval scale, with values being of an integer format. GHQ has been clinically validated, is a screening tool, and the threshold of four deemed valid to represent minor psychiatric morbidity ‘case-ness’ for the UK population, as this low level protects sensitivity[29]. Additionally, it has been used previously in green space and mental health research [30]).Covariates were chosen based on their established associations with visits to heritage and/or mental health [17 31], and examination of full model coefficients confirmed the appropriateness of inclusion of these variables within models. They were sex; age-group (10-year intervals); EIMD income (quintiles); household composition (‘single adult’, ‘single adult with children under 16’, ‘two adults or more’, ‘two adults (or more) with children under 16’); ethnicity (‘White’, ‘Black, Asian and Minority Ethnic (BAME)/mixed’ (numbers did not permit more specific groupings)), highest education qualification (‘degree/other higher degree’, ‘A-levels/GCSEs’, ‘other qualification’, ‘no qualification’), socio-economic status (NS-SEC - ‘managerial/ professional’, ‘intermediate/small employment/own account’, ‘lower supervision/lower technician/semi-routine/routine’, ‘not in (paid) employment’), housing tenure (‘owner occupier’, ‘social renter’, ‘private renter’), and long-standing health condition/mobility (‘none’, ‘condition without limited mobility’, ‘condition with limited mobility’).

Patient and public involvement: We did not involve study participants in the development of the research question, design and implementation of the study or interpretation of the results.

STROBE: Strengthening the Reporting of Observational studies in Epidemiology cross-sectional reporting guidelines were adhered to within this study [32].

See figure one for a flow chart summary of GIS and individual data, including final numbers in analyses. **Figure 1. about here**

For peer review only

Statistical analysis

To explore whether spatial exposure to heritage sites varies by deprivation, we compared mean/median densities of sites (per 1000 population, per square kilometre (km²)), across area income deprivation quintiles, using the Kruskal Wallis test. This test was used as the distribution of these sites indicated that parametric comparisons were not appropriate. We also calculated percentage of heritage sites as 'at risk' across deprivation quintile.

To explore whether spatial exposure to heritage was linked to visiting heritage, we used multilevel logistic regression due to the inclusion of binary outcomes and random effects necessary due to sampling method. Regression was used to determine the association between heritage exposure measures (i.e., presence of sites (none, 1+), sites per population (none, 1-4, 5+) and per area (none, 1-4, 5+)), and whether a respondent had visited a heritage site in the past year (i.e., 'no visits', 'one or more visits yearly')), controlling for individual socio-demographic covariates (listed previously). We undertook further similar modelling to explore whether visits to historic parks/gardens, and scheduled monuments, specifically, were related to exposure to these particular sites respectively.

To explore whether spatial exposure to heritage was linked to mental health, we used multilevel logistic regression to determine the association between heritage exposure measures (as above) and the mental health measure (binary outcome). We controlled for socio-demographic variables, 'visits to heritage' and exposure to 'other green space'. Other green space was included as a confounder to account for potential associations between green space and mental health; such associations were established in prior research [3]. Initially, 'heritage exposure/visits' interactions were included in the models to investigate moderating effects on mental health, with different exposure type/visit interaction run in separate models. If on testing, an interaction was found non-significant, it was removed and models re-run with main effects only. Furthermore, we repeated models with 'built' heritage only (i.e., listed buildings and scheduled monuments) and controlled for 'all green space' (i.e., historic parks/gardens and other green space combined).

In all multilevel models, primary sampling unit (PSU) was set as random effect, weighted by the cross-sectional individual weights supplied by the data providers (see [33] for further information on the PSU). Model results are presented as adjusted odds ratios (OR) and, for independent factors with more than two levels, predicted probabilities (of visits, and of poorer mental health). In addition, post hoc multiple comparison testing was applied. Where applicable, to allow interpretation of results of a model's interaction term, predicted probabilities were presented, and subject to post hoc testing. Regarding missing data, a complete case approach was used, i.e., statistical analysis included only respondents for which there was no missing data on the variables of

For peer review only

interest (n=30,431). Those included and excluded from analysis did not vary in terms sex and education, but did somewhat for age, income, and ethnicity, (included/excluded: 53%/54% female, 36%/36% degree or higher, mean age: 48/43 years, 21%/27% in most deprived areas, 82%/76% white). Multilevel models performed in STATA [34], with a global significance set at 5%.

Results

Heritage exposure varied by area deprivation in a manner dependent on the type of exposure (descriptive information on heritage numbers per quintile available within supplementary table 2). Table 1 shows the distribution of heritage sites per population, and per area/km², by EIMD income quintile. The most deprived areas (Q1) had lower numbers of heritage per 1000 population, while Q4 and Q5 had the highest numbers. This was evident for all three types of heritage analysed together and separately. The pattern for the numbers per area across income deprivation was less clear. The most deprived areas (Q1) had the lowest numbers of listed buildings (mean 9.08), scheduled monuments (0.06) and sites combined (9.23) (see table 1). Q2 had the highest number of listed buildings (12.92), and combined sites (13.10), while Q5 had the highest numbers of scheduled monuments (0.11). Though significant, there was very little variation in density of historic parks/gardens per area across the deprivation quintiles.

For peer review only

Table 1. Distribution of heritage sites (2014) per 1000 population, and per area/km², by EIMD Income (2015) quintile.

	**Listed buildings Mean/median/ (Interquartile range)**		**Historic parks and gardens Mean/median/ (Interquartile range)**		**Scheduled Monuments Mean/median/ (Interquartile range)**		**Overall Mean/median/ (Interquartile range)**	
**EIMD Income**	**per 1000 people**	**per km²**	**per 1000 people**	**per km²**	**per 1000 people**	**per km²**	**per 1000 people**	**per km²**
**1 (most deprived)**	1.79/0.00 (0.00-0.89)	9.08/0.00 (0.00-4.61)	0.02/0.00 (0.00-0.00)	0.09/0.00 (0.00-0.00)	0.03/0.00 (0.00-0.00)	0.06/0.00 (0.00-0.00)	1.83/0.00 (0.00-0.99)	9.23/0.00 (0.00-4.80)
**2**	3.73/0.48 (0.00-2.09)	12.92/0.56 (0.00-6.28)	0.03/0.00 (0.00-0.00)	0.09/0.00 (0.00-0.00)	0.08/0.00 (0.00-0.00)	0.09/0.00 (0.00 - 0.00)	3.84/0.52 (0.00-2.21)	13.10/0.73 (0.00-6.53)
**3**	6.80/0.73 (0.00-4.97)	12.27/1.37 (0.00-5.91)	0.04/0.00 (0.00-0.00)	0.09/0.00 (0.00-0.00)	0.37/0.00 (0.00-0.00)	0.10/0.00 (0.00-0.00)	7.21/0.79 (0.00-5.23)	12.47/1.55 (0.00-6.11)
**4**	11.39/1.57 (0.00-11.19)	9.58/1.72 (0.00-4.56)	0.07/0.00 (0.00-0.00)	0.09/0.00 (0.00-0.00)	0.72/0.00 (0.00-0.00)	0.09/0.00 (0.00-0.00)	12.19/1.72 (0.00-11.82)	9.75/1.89 (0.00-4.76)
**5 (least deprived)**	10.42/1.57 (0.00-9.86)	9.18/1.62 (0.00-4.25)	0.08/0.00 (0.00-0.00)	0.10/0.00 (0.00-0.00)	0.54/0.00 (0.00-0.00)	0.11/0.00 (0.00-0.00)	11.05/1.74 (0.00-10.35)	9.38/1.78 (0.00-4.47)
**England**	6.83/0.64 (0.00-4.19)	10.61/1.13 (0.00-4.99)	0.05/0.00 (0.00-0.00)	0.09/0.00 (0.00-0.00)	0.35/0.00 (0.00-0.00)	0.09/0.00 (0.00-0.00)	7.22/0.66 (0.00-4.41)	10.79/1.31 (0.00-5.20)
**Kruskal Wallis H-value (degrees of freedom)**	H(4) = 2001 P<0.001	H(4) = 788 P<0.001	H(4) = 560 P<0.001	H(4) = 560 P<0.001	H(4) = 1428 P<0.001	H(4) = 1430 P<0.001	H(4) = 2049 P<0.001	H(4) = 714 P<0.001

For peer review only

Table 2 shows the percentage of heritage sites at risk by EIMD income quintile. Around 1.2% (n=4832) of heritage sites (i.e., 2098 listed buildings, 2639 scheduled monuments and 95 historic parks/gardens) were deemed as 'Heritage at Risk' (HAR). Over 2% in total were found in the most deprived fifth of areas, compared to 1% in Q5 (and between 1.1% and 1.4% in the other quintiles). The most deprived areas had the highest percentage of HAR sites across two of the three categories (2.0% of listed buildings, 21.7% of scheduled monuments). In general, the percentage of HAR listed buildings and scheduled monuments increased as areas became more income deprived. The least deprived areas (Q5) exhibited the highest percentage of historic parks/gardens as 'at risk', i.e., 4.1% of sites compared to 3.4% of sites in Q1.

Table 2. Percentage of heritage sites as 'Heritage at Risk' (2016) (HAR) by EIMD income (2015) quintile.

	**Percentage as HAR**			
**EIMD Income**	**Listed buildings**	**Historic Parks/gardens**	**Scheduled Monuments**	**All sites at risk**
**1 (most deprived)**	2.0	3.4	21.7	2.3
**2**	0.8	2.7	16.4	1.1
**3**	0.6	3.8	15.4	1.4
**4**	0.4	3.0	13.2	1.2
**5 (least deprived)**	0.3	4.1	12.6	1.0
**England**	0.6	3.5	13.8	1.2

Of 30,431 respondents, 59.9% had at least one heritage site within their LSOA, and 61% had visited heritage in the past year (n=19,232). Table 3 shows the findings of the multilevel logistic regression analysis as adjusted odds of visiting heritage by exposure to heritage sites. Respondents with at least one heritage site of any type in their neighbourhood (LSOA) were significantly more likely to have visited a heritage site in the past year (Odds Ratio (OR) 1.12 (95% CI: 1.03-1.22)), than those with no sites (p=0.008). Those with five or more heritage sites per 1000 population, or per km², in their LSOA were most likely to have visited a heritage site (OR 1.16 (95% CI: 1.05-1.30) and 1.14 (95% CI: 1.02-1.26) respectively). The presence of scheduled monuments within respondents' neighbourhoods did not increase the probability of visiting monuments specifically, however with an increase in LSOA-level historic parks/gardens per area the likelihood of visiting such sites increased (OR 1.12 (95% CI: 1.04-1.21), p<0.01) (results not shown).

For peer review only

Table 3. Adjusted odds of visiting heritage by measures of exposure to heritage. (Adjusted by household type, sex, age, income deprivation, ethnicity, education, socio-economic status, housing tenure & health condition/mobility)

		**Odds of visiting heritage**	
		**OR (95%CI)**	**p-value**
**Heritage (any)**	**None**	1.00	*0.008*
	**1+**	*1.12 (1.03-1.22)*	
**Heritage (any) per 1000 pop**	**None**	1.00	*0.014*
	**1-4**	*1.10 (1.00-1.20)*	
	**5+**	*1.16 (1.05-1.30)*	
**Heritage (any) per km²**	**None**	1.00	*0.027*
	**1-4**	*1.11 (1.01-1.22)*	
	**5+**	*1.14 (1.02-1.26)*	

When evaluating associations between mental health and exposure to heritage, interaction effects between heritage exposure and heritage visits were found to be associated with mental health. Table 4 presents the predicted probabilities of poorer mental health by heritage exposure and visits to heritage. Post-hoc testing (with Šidák adjustments for multiple comparisons) found the following associations: Amongst those with any heritage, visitors to heritage had a lower predicted probability of distress (0.171 (95% CI: 0.162-0.179)) compared to non-visitors (0.238 (95% CI: 0.225-0.252)) (Šidák adjusted p-value <0.001). This was also true when considering different levels of exposure, i.e. five or more sites per 1000 population (visitors: 0.165 (95% CI: 0.152-0.177), non-visitors: 0.241 (95% CI: 0.219-0.264), (Šidák p=0.008)), or per km² (visitors: 0.171 (95% CI: 0.158-0.184), non-visitors: 0.246 (95% CI: 0.226-0.267), (Šidák p=0.016)). We found similar results when 'built' heritage (i.e., listed buildings/scheduled monuments only) was included as the exposure variable (controlling for any green space). Amongst those with one or more built heritage sites, or with five or more per 1000 population, visitors had a lower predicted probability of distress. In the former, visitors: 0.171 (95% CI 0.163-0.179), non-visitors: 0.237 (95% CI 0.224-0.251) (Šidák p = 0.001) and, in the latter, visitors: 0.165 (95% CI 0.152-0.178), non-visitors: 0.242 (95% CI 0.219-0.265) (Šidák p = 0.009) (results not shown). As main effects, there were no associations between heritage exposure and distress (GHQ-12), regardless of exposure measure used (p-values: 0.055 – 0.224).

For peer review only

Table 4. Predicted probabilities and 95% confidence intervals (CI) of poorer mental health (GHQ12) for the interaction effects of heritage exposure and visits to heritage. (Adjusted by household type, sex, age, income deprivation, ethnicity, education, socio-economic status, housing tenure, health condition/mobility, exposure to other green space & visits to heritage).

		Non-visitors (95% CI)	Visitors (95% CI)	Interaction p-value
**Heritage**	**None**	0.218 (0.204-0.233)	0.184 (0.174-0.195)	*p=0.005*
	**1+**	*0.238 (0.225-0.252)	*0.171 (0.162-0.179)	
**Heritage per 1000 population**	**None**	0.219 (0.205-0.234)	0.185 (0.174-0.196)	*p=0.011*
	**1-4**	0.235 (0.218-0.251)	0.173 (0.163-0.184)	
	**5+**	**0.241 (0.219-0.264)	**0.165 (0.152-0.177)	
**Heritage per area (1 km²)**	**None**	0.219 (0.205-0.234)	0.185 (0.174-0.195)	*p=0.014*
	**1-4**	0.230 (0.213-0.248)	0.169 (0.159-0.179)	
	**5+**	***0.246 (0.226-0.267)	***0.171 (0.158-0.184)	

Post-hoc multiple comparisons: *Difference p<0.001, **Difference p<0.01, ***Difference p<0.05.

Discussion

This is the first study to explore the spatial distribution of designated heritage sites across areas of varying deprivation in England, and associations between exposure and visits, and mental health. We found that, compared to wealthier areas, the poorest areas had fewer sites per population, and, of these sites, a higher proportion were deemed ‘at risk’. Neighbourhood exposure to heritage was associated with visits to heritage; however, the physical presence of neighbourhood heritage, on its own, was not associated with residents’ mental health. The combination of having heritage present in the neighbourhood *and* visiting heritage (whether within the neighbourhood or further afield), was associated with better mental health.

Regarding the social-spatial patterning of heritage, the distribution of heritage varied according to how heritage exposure was measured. The most deprived areas showed the lowest mean heritage sites *per population* (Q1:1.83 per 1000), and the least deprived areas the highest (Q4: 12.2 per 1000, Q5: 11.1 per 1000), however, mean sites *per area* for the least (Q1: 9.2 per km²) and most deprived areas (Q5: 9.4 per km²) were similar. This apparent contradiction in findings between measures likely reflects wealthier areas being on average larger in size. In other words, they do have more heritage sites per population, but these are spread across larger geographical extents (LSOA size increase with increasing wealth, see supplemental table 1). Indeed, greater distance to a local heritage site

For peer review only

may be less of a spatial barrier to those on higher incomes, whom are more likely to own cars [35]. Little existing research investigates spatial distribution of heritage on a national scale. A US city-based study, found that higher income/educated neighbourhoods, had superior access to historical and heritage institutions [36]. The authors described spatial access to historical/heritage institutions as 'elitist' with unequal geographic distribution detrimental to poorer neighbourhoods. In our study, perhaps the spatial bias towards higher income areas is a result of heritage increasing overall area wealth. Indeed, conservation area homes in England benefit from price premiums and greater annual price growth [37], and those with the financial means may pay more to live in homes with historic features [38]. We also report that more heritage sites within poorer areas were 'at risk'. HAR sites are more likely to be in a state of disrepair, less aesthetically pleasing, and potentially unsafe, therefore their higher prevalence in poorer neighbourhoods may result in residents being less likely to engage with heritage. Other existing features of poorer neighbourhoods could compound this, such as higher environmental 'incivility' e.g. graffiti, litter, noise and air pollution [39], and vacant and derelict land [40]. It is conceivable that HAR's greater presence within lower income areas (and greater numbers of heritage sites (overall) per population within wealthier areas), is a result of inequity in the geographical distribution of funding; the Levelling Up paper reports the need for investment in culture and heritage outside London, and within poorer areas [41].

The finding that respondents were more likely to have visited a heritage site in the past year, if they were exposed to local heritage, is comparable to previous evidence on spatial access and attendance at museums or galleries in London [18]. Brook et al. (2016) found that attendance rate variation could not be explained solely by people's individual characteristics and better spatial access was an important contributory factor. Authors concluded that well-resourced locales could provide significant "opportunity structures" for cultural engagement [18]. Certainly better spatial access to other types of environmental features is associated with higher usage, such as sports facilities [42], and green space [43]. We found that those with a higher level of historic parks/gardens locally were more likely to have visited this type of green space in the past year. We cannot say why greater exposure increased the likelihood of heritage visits more generally, or whether local exposure equates to higher local site usage in particular. However, it is feasible that nearby heritage could be a significant 'pull factor' as reduced time and effort may be needed for visits, or visibility of local heritage may contribute to social norms and increase interest in visits, whether near or far.

In terms of a main effect, we found no association between heritage exposure and psychological distress. Associations between heritage engagement and better mental health and wellbeing are established [5-7 9 13 17], however, few studies focus on the mental health benefits of spatial exposure to heritage specifically. The lack of association, in our study, could be attributed to use of

For peer review only

proximity as a 'proxy' for engagement with local heritage, while, in reality, location of visits was unknown. Future surveys could gather data on how far people travel to engage with heritage. We acknowledge that proximity does not indicate use, or awareness of, local heritage. Residents may lack awareness of local neighbourhood features, such as green space [44] or general amenities [45], and this could be true of local heritage. We did find an interaction effect, amongst those with heritage locally; visitors to heritage showed lower psychological distress. A European-based study looked at associations between neighbourhood green/blue space and mental health [46], and found that although people in highly green/coastal areas experienced better mental health, this relationship did not remain when controlling for recreational visits to these areas. Visit frequency appeared to mediate associations between green/blue space and wellbeing. Authors reasoned that better mental health amongst those in the greenest/bluest areas could be a result of such environmental qualities encouraging visits. Similarly, we found that a combination of proximal heritage, and visits to heritage, was associated with better mental health; higher spatial exposure appeared to provide a pathway to encourage visits. Our findings contribute to discussions about which type of neighbourhood exposure is key in associations with mental health. Many neighbourhood effects' studies define 'exposure' as 'residential proximity', e.g. green space [47]. It is valuable to examine both spatial exposure, and direct contact/engagement, when considering mental health. The beneficial association between greenspace and mental health has been well researched, with theories of benefit based on visualising the structure of greenspace, its colours and its landscapes, to engage the human brain, and work to reduce stress, restore attention, and enhance wellbeing [48]. Less is known about how 'bricks and mortar' heritage influences mental health. We found that, regardless of green space exposure, those with built heritage in their local area, who had visited heritage, showed lower distress. Historic buildings are considered to improve neighbourhood quality with older buildings creating feelings of grandeur and permanence [38], and exposure to urban environments with historic features, has been perceived more restorative, attractive and interesting than those without [49 50]. Viewing heritage appears to engage the senses, and places considered 'special' may stimulate the brain cells that manage emotion [51]. Residents who live in areas with higher proportions of listed buildings, or who regard a building as 'special' within their local area, have a greater 'sense of place' [10] which, in turn, can be associated with better mental health [52]. Regarding local heritage as 'special' could thus be part of the pathway connecting heritage to wellbeing.

Strengths and limitations

Our research exhibited a number of strengths. We used a sample of adults from across the whole of England, weighted to ensure representativeness to the wider population. We explored associations

For peer review only

between exposure to heritage, and to visits, and to mental health, within relatively small areas across the whole of England, while other papers did not incorporate spatial data or nation-wide analyses. We explored whether mental health benefits of 'built' heritage remained regardless of the presence of green space in the local neighbourhood, providing a unique strand to this research. Regarding limitations, this study was cross-sectional; we cannot assume that key variables demonstrate causal associations, and that findings are generalisable within other contexts. We acknowledge that use of LSOA-level analysis may lead to statistical bias from using arbitrarily classified units to report spatial patterning [53]. Our study may be limited in that we do not know whether UKHLS respondents visit heritage sites within their local area or further afield. We may be limited by the simplified nature of the UKHLS questions on visits to heritage, e.g., those visiting a 'A city or town with historic character', may have done so for another purpose, i.e., for every-day activities related to employment, education, food shopping etc. Unfortunately, more detailed heritage visits data that can be linked to spatial data is not available. Doing every-day activities within a heritage environment may be associated with better mental health, however researching this is beyond the scope of our current study. Our study explores spatial exposure to heritage only, and does not incorporate other factors, which may promote or inhibit accessibility, such as financial access, e.g., admittance fees. Although heritage may be free to view. We acknowledge the potential for a small number of HAR sites to be missing from the at risk register; lack of resources may result in systematic surveys of different HAR types being undertaken at different times [54]. Additionally, we acknowledge that individuals with poorer mental health may visit heritage sites less frequently due to barriers relating to motivations and opportunities.

Policy implications

Our findings of inequities in heritage exposure, as a barrier to heritage engagement, are directly relevant to the UK Government's levelling up agenda. The bill recommends ensuring protection to existing heritage, tackling disparities in heritage access, and improving cultural investment outside London [41]. Going forward the levelling up agenda must address inequalities in heritage exposure as part of a pathway to improve societal mental health. Methods to increase exposure within areas with fewer heritage sites could include better options for public transport, e.g. subsidised transport to areas with better exposure [36]. Exposure could be improved via investment in heritage in need of reinvigoration, such as at risk sites, and such sites can be improved via heritage volunteering schemes which, additionally, provide various social, psychological and health benefits to volunteers [55]. Increasing heritage engagement opportunities must consider both geographical and socio-cultural factors [18]. Policy objectives to promote heritage engagement across all cultural groups should seek to redress potentially lower levels of interest among specific groups, such as poorer

For peer review only

households [17]. Heritage organisations could expand 'public outreach' activities to neighbourhoods and communities with lower exposure [36], or with lower levels of heritage awareness, and compensate for fewer heritage assets in deprived areas, through increased awareness of what heritage *is* there.

Conclusion

We demonstrated that neighbourhoods with higher levels of income deprivation in England had lower levels of heritage exposure. Exposure to local heritage increased the likelihood of visiting heritage in general, and exposure to any heritage, or built heritage specifically, was positively associated with mental health, but only amongst those who had visited heritage in the past year. Our findings indicate that both spatial exposure and engaging with heritage through visiting are key. There is much to be gained from improving the structure of exposure. Formulation of strategies for schemes to tackle inequality in exposure in poorer neighbourhoods is necessary. Schemes could include working with communities and areas to improve access, knowledge, and awareness of heritage, and providing investment in 'at risk' heritage and currently non-accessible sites. Such schemes could promote local heritage as 'special', or distinctive in a positive way, to improve heritage engagement, and ultimately provide benefits to mental health and well-being.

Acknowledgements: We wish to thank Neil Guiden (Data & Analysis Manager) at Historic England for supplying spatial data. We wish to acknowledge the UKHLS participants for their involvement in the study and the UK Data Service for supplying all social survey data used.

Authors contributors: LMac, EG, LM, and RM proposed the research questions. LMac led the project, created GIS variables, and drafted all versions of the article. NN undertook statistical analysis and interpretation. All authors critically revised the article, read and approved final draft.

Funding: LMac, NN and RM are funded by the UK Medical Research Council (MRC) Places and Health Programme (MC_UU_00022/4) and the Chief Scientist Office (CSO) (SPHSU2019) at the MRC/CSO Social and Public Health Sciences Unit, University of Glasgow, UK.

Competing interests: The authors declare no conflict of interest.

Patient consent for publication: Not required.

Ethics approval: The University of Essex Ethics Committee has approved all data collection on Understanding Society. The UKHLS main study gained consent for all data linkages (except to health records) and respondents aged 16 or over provided informed and written consent to participate.

Provenance and peer review: Not commissioned; externally peer reviewed.

For peer review only

Data availability statement: Data may be obtained from a third party and are not publicly available.

Supplemental material: This content has been supplied by the authors.

Open access: This is an open access article distributed in accordance with the Creative Commons Attribution 4.0 Unported (CC BY 4.0) license, which permits others to copy, redistribute, remix, transform and build upon this work for any purpose, provided the original work is properly cited, a link to the licence is given, and indication of whether changes were made. See: https://creativecommons.org/licenses/by/ 4.0/.

ORCID: Laura Macdonald https://orcid.org/0000-0002-0593-8079

Figure 1. Flow chart summary of GIS and individual data used.

References

1. Jimenez MP, DeVille NV, Elliott EG, *et al.* Associations between nature exposure and health: A review of the evidence. *International Journal of Environmental Research and Public Health* 2021;18:4790.
2. Twohig-Bennett C, Jones A. The health benefits of the great outdoors: A systematic review and meta-analysis of greenspace exposure and health outcomes. *Environmental research* 2018;166:628-37.
3. Ward Thompson C, Silveirinha de Oliveira E, Wheeler BW, *et al.* Urban Green Spaces and Health, A Review of Evidence. Copenhagen: World Health Organisation, 2016.
4. Historic England. About The List. 2022. https://historicengland.org.uk/listing/the-list/about-the-list/ [Accessed 27 June 2022].
5. Brown JL, MacDonald R, Mitchell R. Are People Who Participate in Cultural Activities More Satisfied with Life? *Social Indicators Research* 2015;122:135-46.
6. Fujiwara D, Cornwall T, Dolan P. Heritage and Wellbeing. London: English Heritage, 2014.
7. Leadbetter C, O'Connor N. Healthy Attendance? The Impact of Cultural Engagement and Sports Participation on Health and Satisfaction with Life in Scotland. Edinburgh: Scottish Government, 2013.
8. Lakey J, Smith N, Oskala A, *et al.* Culture, sport and well-being. Findings from the Understanding Society Youth Survey. London: NatCen Social Research, 2017.
9. Mak HW, Coulter R, Fancourt D. Associations between community cultural engagement and life satisfaction, mental distress and mental health functioning using data from the UK Household Longitudinal Study (UKHLS): are associations moderated by area deprivation? *BMJ open* 2021;11:e045512.
10. Bradley DP, Coombes M, Tranos E, *et al.* Sense of place and social capital and the historic built environment: Report of research for English Heritage. Newcastle: Newcastle University and Bradley Research and Consulting, 2009.
11. Harris PB, Werner CM, Brown BB, *et al.* Relocation and privacy regulation: A cross-cultural analysis. *Journal of environmental psychology* 1995;15:311-20.
12. Rollero C, De Piccoli N. Does place attachment affect social well-being? *European Review of Applied Psychology* 2010;60:233-38.

For peer review only

13. Darvill T, Heaslip V, Barrass K. Heritage and well-being: Therapeutic places past and present. In: Darvill T, Barrass K, Drysdale L, et al., eds. Historic Landscapes and Mental Well-being. Oxford: Archaeopress, 2019:17.
14. Grossi E, Tavano Blessi G, Sacco PL. Magic moments: determinants of stress relief and subjective wellbeing from visiting a cultural heritage site. *Culture, Medicine, and Psychiatry* 2019;43:4-24.
15. Sofaer J, Davenport B, Sørensen MLS*, et al.* Heritage sites, value and wellbeing: learning from the COVID-19 pandemic in England. *International Journal of Heritage Studies* 2021;27:1117-32.
16. National Statistics. Taking Part 2019/20 - Heritage. 2020. https://www.gov.uk/government/statistics/taking-part-201920-heritage [Accessed 08 February 2022].
17. Lakey J, Smith N, Oskala A*, et al.* Culture, sport and well-being. Findings from the Understanding Society Adult Survey. London: NatCen Social Research, 2017.
18. Brook O. Spatial equity and cultural participation: how access influences attendance at museums and galleries in London. *Cultural Trends* 2016;25:21-34.
19. OCSI. 'Left behind' Neighbourhoods: Community data dive. 2021. https://www.appg-leftbehindneighbourhoods.org.uk/wp-content/uploads/2021/06/APPG-Community-Data-Dive-Report-for-APPG-S7.pdf [Accessed 09 February 2022].
20. HM Government. Levelling Up the United Kingdon. London: Her Majesty's Stationery Office, 2022.
21. Office for National Statistics. Census geography. 2021. https://www.ons.gov.uk/methodology/geography/ukgeographies/censusgeography [Accessed 09 May 2022].
22. UK Government. English indices of deprivation 2015. 2015. https://www.gov.uk/government/statistics/english-indices-of-deprivation-2015 [Accessed 20 October 2021].
23. Office for National Statistics. Lower layer Super Output Area population estimates. 2021. https://www.ons.gov.uk/peoplepopulationandcommunity/populationandmigration/populationestimates/datasets/lowersuperoutputareamidyearpopulationestimates [Accessed 20 October 2021].
24. Historic England. What is the Heritage at Risk Programme? 2022. https://historicengland.org.uk/advice/heritage-at-risk/types/ [Accessed 21 March 2022].
25. Ordnance Survey. OS Open Greenspace. 2021. https://www.ordnancesurvey.co.uk/business-government/products/open-map-greenspace.
26. Office for National Statistics. Lower Layer Super Output Area (LSOA) boundaries. 2016. https://data.gov.uk/dataset/fa883558-22fb-4a1a-8529-cffdee47d500/lower-layer-super-output-area-lsoa-boundaries.
27. Kaminska O, ., Lynn P. Weighting and Sample Representation. London: Institute for Social and Economic Research, University of Essex, 2019.
28. Goldberg DP. User's guide to the General Health Questionnaire. Windsor, 1988.
29. Goldberg DP, Oldehinkel T, Ormel J. Why GHQ threshold varies from one place to another. *Psychological medicine* 1998;28:915-21.
30. Astell-Burt T, Mitchell R, Hartig T. The association between green space and mental health varies across the lifecourse. A longitudinal study. *Journal of Epidemiology and Community Health* 2014;68:578-83.
31. Mak HW, Coulter R, Fancourt D. Associations between neighbourhood deprivation and engagement in arts, culture and heritage: evidence from two nationally-representative samples. *BMC public health* 2021;21:1-10.
32. Von Elm E, Altman DG, Egger M*, et al.* The Strengthening the Reporting of Observational Studies in Epidemiology (STROBE) statement: guidelines for reporting observational studies. *Annals of internal medicine* 2007;147:573-77.

For peer review only

33. UKHLS. Clustering and stratification. 2022. https://www.understandingsociety.ac.uk/documentation/mainstage/user-guides/main-survey-user-guide/clustering-and-stratification [Accessed 11 January 2023].
34. StataCorp. Stata Statistical Software. College Station, Texas: StataCorp LLC, 2021.
35. Department for Transport. Travel by vehicle availability, income, ethnic group, household type, mobility status and NS-SEC. 2020. https://www.gov.uk/government/statistical-data-sets/nts07-car-ownership-and-access#full-publication-update-history [Accessed 31 May 2022].
36. Moldavanova AV, Meloche L, Thompson TL. Understanding the geography of access to cultural amenities: The case of Metropolitan Detroit. *Journal of Urban Affairs* 2021:1-26.
37. Ahlfeldt GM, Holman N, Wendland N. An assessment of the effects of conservation areas on value. London: London School of Economics and Political Science, 2012.
38. Maeer G, Robinson A, Hobson M. Values and benefits of heritage A research review. London: Heritage Lottery Fund, 2016.
39. Braubach M, Fairburn J. Social inequities in environmental risks associated with housing and residential location—a review of evidence. *European journal of public health* 2010;20:36-42.
40. Macintyre S, Macdonald L, Ellaway A. Do poorer people have poorer access to local resources and facilities? The distribution of local resources by area deprivation in Glasgow, Scotland. *Social science & medicine* 2008;67:900-14.
41. The Heritage Alliance. Levelling Up White Paper Summary for Heritage. London: The Heritage Alliance, 2022.
42. Giles-Corti B, Donovan RJ. The relative influence of individual, social and physical environment determinants of physical activity. *Social Science & Medicine* 2002;54:1793–812.
43. Pyky R, Neuvonen M, Kangas K*, et al.* Individual and environmental factors associated with green exercise in urban and suburban areas. *Health & place* 2019;55:20-28.
44. Macintyre S, Macdonald L, Ellaway A. Lack of agreement between measured and self-reported distance from public green parks in Glasgow, Scotland. *International Journal of Behavioral Nutrition and Physical Activity* 2008;5:1-8.
45. Macdonald L, Kearns A, Ellaway A. Do residents' perceptions of being well-placed and objective presence of local amenities match? A case study in West Central Scotland, UK. *BMC Public Health* 2013;13:1-11.
46. White MP, Elliott LR, Grellier J*, et al.* Associations between green/blue spaces and mental health across 18 countries. *Scientific reports* 2021;11:1-12.
47. Bratman GN, Hamilton JP, Daily GC. The impacts of nature experience on human cognitive function and mental health. *Annals of the New York Academy of Sciences* 2012;1249:118–36.
48. Markevych I, Schoierer J, Hartig T*, et al.* Exploring pathways linking greenspace to health: Theoretical and methodological guidance. *Environmental research* 2017;158:301-17.
49. Bornioli A, Parkhurst G, Morgan PL. Psychological wellbeing benefits of simulated exposure to five urban settings: an experimental study from the pedestrian's perspective. *Journal of transport & health* 2018;9:105-16.
50. Bornioli A, Parkhurst G, Morgan PL. The psychological wellbeing benefits of place engagement during walking in urban environments: A qualitative photo-elicitation study. *Health & place* 2018;53:228-36.
51. National Trust. Why Places Matter to People Research Report. Wiltshire: National Trust, 2018.
52. Williams A, Kitchen P. Sense of Place and Health in Hamilton, Ontario: A Case Study. *Social indicators research* 2012;108:257-76.
53. Openshaw S. The Modifiable Areal Unit Problem. Norwich, England: Geobooks, 1984.
54. Historic England. Heritage at Risk Statistics: Method Statement. London: Historic England, 2021.
55. Lewis C, Siriwardena N, Laparidou D*, et al.* Wellbeing in Volunteers on Heritage at Risk Projects. London: Historic England, 2021.

For peer review only - http://bmjopen.bmj.com/site/about/guidelines.xhtml

Figure 1. Flow chart summary of GIS and individual data used

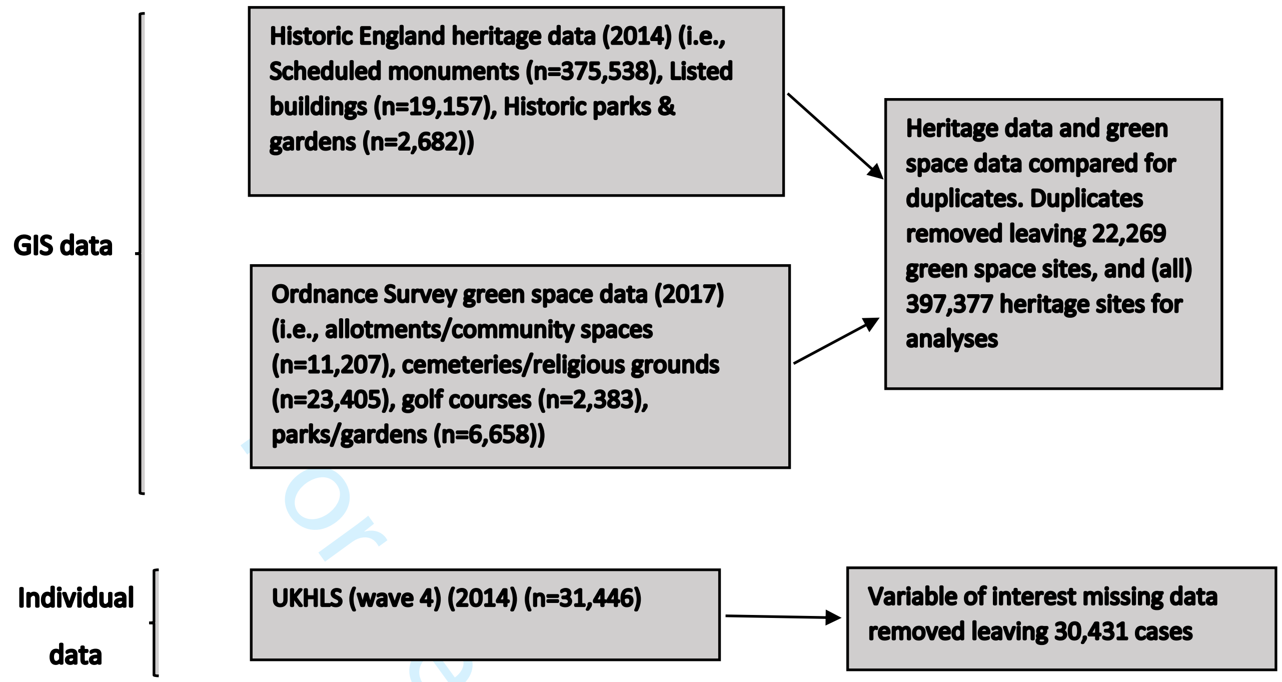

For peer review only

Supplemental table 1. EIMD Income Quintile descriptive – population, geographical area, heritage.

EIMD Income	Population (Mean, SD)	Area km² (Mean, SD)	Listed buildings (N)	Historic Parks/ Gardens (N)	Scheduled Monuments (N)	All sites (N)
**1 (most deprived)**	11,075,909 (1,686, 331.36)	3,428 (0.52, 1.19)	19,880	203	277	20,360
**2**	11,149,857 (1,697, 362.87)	7,547 (1.15, 3.71)	41,189	292	913	42,394
**3**	11,016,312 (1,677, 360.18)	26,114 (3.98, 12.42)	75,084	470	4,170	79,724
**4**	10,820,663 (1,647, 355.98)	50,252 (7.65, 20.69)	125,462	790	7,904	13,4156
**5 (least deprived)**	10,723,586 (1,632, 407.47)	43,089 (6.56, 15.89)	113,923	927	5,893	120,743
**Total**	54,786,327 (1,668, 365.19)	130,432 (3.97, 13.34)	375,538	2,682	19,157	397,377

Supplemental table 2. Complete case sample descriptive (percentage by each category)

(base n=30420)

**Age**	<20 years old	6.1
	20-29 years old	12.6
	30-39 years old	15.9
	40-49 years old	18.9
	50-59 years old	16.9
	60-69 years old	15.3
	70+ years old	14.4
	Missing	0
**Sex**	Male	44.6
	Females	55.4
	Missing	0

For peer review only

**EIMD income**	Q1 (most deprived)	20.5
	Q2	19.3
	Q3	20.6
	Q4	20.7
	Q5 (least deprived)	18.9
	Missing	0
**Ethnicity**	White	82.5
	Other	17.5
	Missing	21
**Education**	Degree or higher	36.4
	GCSEs/A-levels	41.8
	Other qualification	9.6
	No qualification	12.3
	Missing	54
**Job type (NS-SEC)**	Managerial, admin & professional	24.7
	Intermediate occupations	13.7
	Routine & manual occupations	19.8
	Not in paid employment	41.9
	Missing	341
**Housing tenure**	Owner occupier	71.3
	Social renter	16.7
	Private renter	12.1
	Missing	499
**Long-term illness/mobility**	No long-term illness or mobility issue	53.0
	Long-term illness	14.8
	Long-term illness and mobility issue	32.2
	Missing	11

For peer review only

**Marital/cohabitating status**	Single	21.8
	Married/cohabiting	63.7
	Divorced/separated	8.6
	Widowed	5.9
	Missing	70

For peer review only

Reporting checklist for cross sectional study.

Based on the STROBE cross sectional guidelines.

Instructions to authors

Complete this checklist by entering the page numbers from your manuscript where readers will find each of the items listed below.

Your article may not currently address all the items on the checklist. Please modify your text to include the missing information. If you are certain that an item does not apply, please write "n/a" and provide a short explanation.

Upload your completed checklist as an extra file when you submit to a journal.

In your methods section, say that you used the STROBE cross sectionalreporting guidelines, and cite them as:

von Elm E, Altman DG, Egger M, Pocock SJ, Gotzsche PC, Vandenbroucke JP. The Strengthening the Reporting of Observational Studies in Epidemiology (STROBE) Statement: guidelines for reporting observational studies.

For peer review only

		Reporting Item	Page Number
**Title and abstract**			
Title	#1a	Indicate the study’s design with a commonly used term in the title or the abstract	2
Abstract	#1b	Provide in the abstract an informative and balanced summary of what was done and what was found	2

Introduction

Background / rationale	#2	Explain the scientific background and rationale for the investigation being reported	3-5
Objectives	#3	State specific objectives, including any prespecified hypotheses	5

Methods

Study design	#4	Present key elements of study design early in the paper	5-9
Setting	#5	Describe the setting, locations, and relevant dates, including periods of recruitment, exposure, follow-up, and data collection	5-9
Eligibility criteria	#6a	Give the eligibility criteria, and the sources and methods of selection of participants.	8
	#7	Clearly define all outcomes, exposures, predictors, potential confounders, and effect modifiers. Give diagnostic criteria, if applicable	5-9
Data sources / measurement	#8	For each variable of interest give sources of data and details of methods of assessment (measurement). Describe comparability of assessment methods if there is more than one group. Give information separately for for exposed and unexposed groups if applicable.	5-9
Bias	#9	Describe any efforts to address potential sources of bias	5-9

For peer review only

Study size	#10	Explain how the study size was arrived at	5-9
Quantitative variables	#11	Explain how quantitative variables were handled in the analyses. If applicable, describe which groupings were chosen, and why	5-9
Statistical methods	#12a	Describe all statistical methods, including those used to control for confounding	8-9
Statistical methods	#12b	Describe any methods used to examine subgroups and interactions	8-9
Statistical methods	#12c	Explain how missing data were addressed	9
Statistical methods	#12d	If applicable, describe analytical methods taking account of sampling strategy	n/a
Statistical methods	#12e	Describe any sensitivity analyses	n/a
**Results**			
Participants	#13a	Report numbers of individuals at each stage of study—eg numbers potentially eligible, examined for eligibility, confirmed eligible, included in the study, completing follow-up, and analysed. Give information separately for for exposed and unexposed groups if applicable.	n/a
Participants	#13b	Give reasons for non-participation at each stage	n/a
Participants	#13c	Consider use of a flow diagram	n/a

For peer review only

Descriptive data	#14a	Give characteristics of study participants (eg demographic, clinical, social) and information on exposures and potential confounders. Give information separately for exposed and unexposed groups if applicable.	(supplemental table)
Descriptive data	#14b	Indicate number of participants with missing data for each variable of interest	(supplemental table)
Outcome data	#15	Report numbers of outcome events or summary measures. Give information separately for exposed and unexposed groups if applicable.	n/a
Main results	#16a	Give unadjusted estimates and, if applicable, confounder-adjusted estimates and their precision (eg, 95% confidence interval). Make clear which confounders were adjusted for and why they were included	9-12
Main results	#16b	Report category boundaries when continuous variables were categorized	7
Main results	#16c	If relevant, consider translating estimates of relative risk into absolute risk for a meaningful time period	n/a
Other analyses	#17	Report other analyses done—e.g., analyses of subgroups and interactions, and sensitivity analyses	9-10

Discussion

For peer review only

Key results	#18	Summarise key results with reference to study objectives	13
Limitations	#19	Discuss limitations of the study, taking into account sources of potential bias or imprecision. Discuss both direction and magnitude of any potential bias.	15
Interpretation	#20	Give a cautious overall interpretation considering objectives, limitations, multiplicity of analyses, results from similar studies, and other relevant evidence.	13-16
Generalisability	#21	Discuss the generalisability (external validity) of the study results	15
**Other Information**			
Funding	#22	Give the source of funding and the role of the funders for the present study and, if applicable, for the original study on which the present article is based	17

Notes:

- 14a: 15 (supplementary table)
- 14b: 15 (supplementary table)
- 16a: 9-10 (and tables) The STROBE checklist is distributed under the terms of the Creative Commons Attribution License CC-BY. This checklist was completed on 26. July 2022 using https://www.goodreports.org/, a tool made by the EQUATOR Network in collaboration with Penelope.ai

For peer review only
